# Quantitation and Comparison of Phenotypic Heterogeneity Among Single Cells of Monoclonal Microbial Populations

**DOI:** 10.3389/fmicb.2019.02814

**Published:** 2019-12-20

**Authors:** Federica Calabrese, Iryna Voloshynovska, Florin Musat, Martin Thullner, Michael Schlömann, Hans H. Richnow, Johannes Lambrecht, Susann Müller, Lukas Y. Wick, Niculina Musat, Hryhoriy Stryhanyuk

**Affiliations:** ^1^Department of Isotope Biogeochemistry, Helmholtz Centre for Environmental Research-UFZ, Leipzig, Germany; ^2^le-tex Publishing Services GmbH, Leipzig, Germany; ^3^Department of Environmental Microbiology, Helmholtz Centre for Environmental Research-UFZ, Leipzig, Germany; ^4^Institute of Biosciences, TU-Bergakademie Freiberg, Freiberg, Germany

**Keywords:** phenotypic heterogeneity, single-cell resolution, SIP–nanoSIMS, anabolic activity, flow cytometry, multimodality, heterogeneity quantitation, Zipf's law

## Abstract

Phenotypic heterogeneity within microbial populations arises even when the cells are exposed to putatively constant and homogeneous conditions. The outcome of this phenomenon can affect the whole function of the population, resulting in, for example, new “adapted” metabolic strategies and impacting its fitness at given environmental conditions. Accounting for phenotypic heterogeneity becomes thus necessary, due to its relevance in medical and applied microbiology as well as in environmental processes. Still, a comprehensive evaluation of this phenomenon requires a common and unique method of quantitation, which allows for the comparison between different studies carried out with different approaches. Consequently, in this study, two widely applicable indices for quantitation of heterogeneity were developed. The heterogeneity coefficient (HC) is valid when the population follows unimodal activity, while the differentiation tendency index (DTI) accounts for heterogeneity implying outbreak of subpopulations and multimodal activity. We demonstrated the applicability of HC and DTI for heterogeneity quantitation on stable isotope probing with nanoscale secondary ion mass spectrometry (SIP–nanoSIMS), flow cytometry, and optical microscopy datasets. The HC was found to provide a more accurate and precise measure of heterogeneity, being at the same time consistent with the coefficient of variation (CV) applied so far. The DTI is able to describe the differentiation in single-cell activity within monoclonal populations resolving subpopulations with low cell abundance, individual cells with similar phenotypic features (e.g., isotopic content close to natural abundance, as detected with nanoSIMS). The developed quantitation approach allows for a better understanding on the impact and the implications of phenotypic heterogeneity in environmental, medical and applied microbiology, microbial ecology, cell biology, and biotechnology.

## Introduction

Ecosystems and microbial communities have traditionally been studied as an assembly of different monoclonal populations. Each population has been considered to be composed of genetically identical cells performing the same metabolic function. However, techniques with single-cell resolution were developed over the past decades, allowing us to narrow down the vision at the level of individual cells (Brehm-Stecher and Johnson, 2004; Pumphrey et al., 2009; Vasdekis and Stephanopoulos, 2015; Gao et al., 2016). This facilitates the understanding of monoclonal population's physiology, strengthening the concept that genetically identical cells can show cell-to-cell variability even when sharing the same environmental and nutritional conditions and phenomenon, generically known as phenotypic heterogeneity (Avery, 2006; Ackermann, 2015; Davis Kimberly and Isberg Ralph, 2016). By definition, the phenotype is every observable feature of an organism originating from the complex interaction between genotype, cellular biochemical mechanisms, and environment. Although a univocal definition of phenotypic heterogeneity is currently missing in literature, this usually embraces all intrinsic and extrinsic cellular noise (Simpson et al., 2009) as well as environment-induced differences arising between single cells belonging to a monoclonal isogenic population under homogeneous environmental conditions. Phenotypic heterogeneity arises from stochastic effects in random molecular and biochemical processes (Elowitz et al., 2002; Kærn et al., 2005; Kussell and Leibler, 2005; Kiviet et al., 2014), inequalities in gene expression, and transcriptional regulatory networks (Newman et al., 2006; Maheshri and O'shea, 2007; Fraser and Kærn, 2009) as well as constitutive single-cell features like cell cycle or cell aging (Sumner and Avery, 2002; Levy et al., 2012). All these effects contribute to the so-called cellular noise (Avery, 2006; Samoilov et al., 2006; Tsimring, 2014), which is extensively used in literature referring to cell-to-cell variability due to molecular mechanisms, regulatory pathways, and genetic cues (Balázsi et al., 2011; Levchenko and Nemenman, 2014). The unequal segregation of DNA hyper-structures transmitted to the daughter cells during division and the variation in populations' growth rate lead to the diversification in substrate assimilation by single cells in batch and chemostat cultures (Kopf Sebastian et al., 2015; Gangwe Nana et al., 2018). Metabolic/functional diversifications help microbial populations to cope better with stresses and fluctuations in their surrounding environment (Avery, 2006; Ackermann, 2015; Bódi et al., 2017). Indeed, differences at the metabolic level occur when cells respond to niche perturbations with different metabolic strategies gaining new ecological functions from which the whole population benefits (West and Cooper, 2016; Bódi et al., 2017). The environment affects considerably the metabolic fluxes inside bacterial cells via activation of different regulatory pathways of the central carbon metabolism, thus leading to different phenotypes within an isogenic population (Kotte et al., 2014). Yeast cells reveal multiple metabolic states under certain environmental conditions, regulating dynamically the glycolysis pathway and thus preventing metabolic imbalances (Van Heerden et al., 2014). The occurrence of cell-to-cell differences in metabolic traits results from an interplay of ecological, intracellular and extracellular factors, usually referred to as metabolic heterogeneity (Takhaveev and Heinemann, 2018). Thus, the term phenotypic heterogeneity encompasses all aspects of cell-to-cell variability including epigenetic, regulatory, metabolic, physical, or physiological aspects observed at the single-cell level.

A challenge in the resolution of single-cell phenotypic features as well as in the tracking of intracellular metabolic fluxes remains the detection limits of the applied experimental techniques and their throughput (Takhaveev and Heinemann, 2018). Nanoscale secondary ion mass spectrometry (nanoSIMS) provides high lateral resolution for single microbial cell imaging together with a mass resolving power (MRP) sufficient for tracking the assimilation of isotope-labeled substrates (Lechene et al., 2006). A combination of stable isotope probing (SIP) with nanoSIMS (SIP–nanoSIMS) has been applied to shed light on single-cell metabolic activity within microbial populations in environmental setups as well as under laboratory conditions (Musat et al., 2008; Pumphrey et al., 2009; Pett-Ridge and Weber, 2012; Sheik et al., 2015; Zimmermann et al., 2015; Jiang et al., 2016; Schreiber et al., 2016; Nikolic et al., 2017; Nuñez et al., 2017). Studies applying SIP–nanoSIMS have been focused on assimilation activity in the frame of specific metabolic functions, considering exclusively the heterogeneity in terms of isotopic enrichment at the single-cell level. Metabolic heterogeneity in monoclonal population has been shown to increase under limitation of nutrients and/or electron donors (Zimmermann et al., 2015, 2018; Schreiber et al., 2016); nutritional and temperature upshifts as well as carbon-source competition enhance metabolic specialization among monoclonal cells (Sheik et al., 2015; Nikolic et al., 2017; Simşek and Kim, 2018). The above-mentioned studies have quantified the uptake of isotope-labeled substrates normalizing it with output from bulk measurements and applying empirical assimilation expressions. It is difficult to compare the results on metabolic activity from different studies, even when derived from SIP–nanoSIMS experiments, if a unified approach for quantitation of single-cell assimilation is not followed. A SIP–nanoSIMS-based approach has recently introduced the “relative assimilation” (*K*_*A*_) as a measure to quantify the single-cell assimilation activity (Stryhanyuk et al., 2018). However, being exceptionally powerful in quantitation of isotopic composition at the single-cell level, nanoSIMS-based approaches require a long time to acquire chemical maps for a limited number of single cells (~1–10^2^), implying thus a considerable limitation in the method throughput.

Flow cytometry and fluorescence time-lapse microscopy have also been applied to study phenotypic heterogeneity (Davey and Kell, 1996; Müller and Babel, 2003; Balaban et al., 2004; Nikolic et al., 2013, 2017; Lieder et al., 2014; Pratt et al., 2019). The output of these methods' application comprises multidimensional datasets providing information on, for example, gene expression, physiology, metabolism, and morphology of individual cells (Davey and Kell, 1996; Müller and Babel, 2003; Balaban et al., 2004; Nikolic et al., 2013, 2017; Lieder et al., 2014; Pratt et al., 2019). Notably, the data are acquired within a short time while analyzing thousands of cells (~10^3^-10^5^). With single-cell resolution and high sensitivity, flow cytometry is however not an imaging technique, and it cannot be applied to resolve intracellular compartments and single cells in spatial arrangements.

Physical properties of single cells (e.g., their morphology, intracellular and extracellular features, and viability) and the 3-D structure of their arrangement can be characterized with optical, atomic force, or electron/ion probe microscopy techniques (Hawkes and Spence, 2007; Hlawacek and Gölzhäuser, 2016). The microscopy imaging delivers information on single-cell phenotypes (geometry, size, volume, etc.), showing that the size of individual cells differs from the population average, which in turn is modulated by the environment and the growth conditions (Grover and Woldringh, 2001; Taheri-Araghi et al., 2015; Nikolic et al., 2017; Westfall and Levin, 2018).

To our knowledge, a general approach to quantify phenotypic heterogeneity has not been developed to date. Nowadays, the interest on phenotypic heterogeneity is increasing due to its implications in medical microbiology, microbial ecology, and biotechnology. Phenotypic heterogeneity has been shown to have an impact on medical care, concerning antibiotic resistance, treatment persistence, and biofilm formation (Turner et al., 2000; Sumner and Avery, 2002; Balaban et al., 2004; Grote et al., 2015; Dhar et al., 2016; Sadiq et al., 2017; Van Den Bergh et al., 2017) but also in biomedical research for drug discovery, cancer therapy, and diagnostics due to the variable effectiveness of care treatments on different phenotypes (Almendro et al., 2013; Gough et al., 2017). Understanding cell-to-cell heterogeneity in bioprocessing is currently a big challenge due to its implications in processes' performance and therefore product yield in large-scale production (Delvigne et al., 2014, 2017). Hence, the quantitation of heterogeneity becomes relevant for an efficient process optimization in every field of biotechnology.

Phenotypic heterogeneity has been so far expressed with the coefficient of variation (CV) for its comparison under different experimental conditions (Grover and Woldringh, 2001; Kopf Sebastian et al., 2015; Schreiber et al., 2016; Nikolic et al., 2017). The CV is calculated as the ratio of standard deviation over the arithmetic mean value, and its application as a measure of heterogeneity implies therefore a normal distribution of single cells in their activity or function. However, this is not always the case for a heterogeneous population, in which a large dispersion of data from the centroid is observed and the distribution shape is often asymmetrical (skewed). Measures of dispersion, such as coefficient of quartile deviation (CQD) (Bonett, 2006) or coefficient of dispersion (COD) (Bonett and Seier, 2006), are likewise used to calculate the scattering of the data around the centroids, but they are affected differently by fluctuations (outliers) of observations in a dataset. Additionally, there are currently no indices accounting for one of the distinctive features of phenotypic heterogeneity, that is, the uprising of clonal subpopulations (multimodality phenomenon) with different metabolic activities (Arnoldini et al., 2014; Li et al., 2018; Simşek and Kim, 2018). An attempt to quantify the growth-related heterogeneity of microbial populations has been recently done in a flow cytometry experiment (Heins et al., 2019), where authors considered the slope of cumulative distribution function in combination with skewness, peak width, and CV. The suggested combination of distribution parameters may represent an average or describe a most abundant subpopulation but does not provide a robust measure of heterogeneity for a multimodal distribution.

By extending the concept of CV and considering the distribution of single-cell anabolic activity measured in *K*_*A*_ (Stryhanyuk et al., 2018), we developed a novel approach for heterogeneity expression which returns the “heterogeneity coefficient” (HC) involving the correction for the counting statistics error (CSE) coming from nanoSIMS analysis. Since neither the CV nor the HC accounts for the outcome of subpopulations, the challenge of quantitating heterogeneity in multimodal single-cell activity had to be tackled. With this purpose, a modification of power Zipf's (1935) law describing the rank–frequency distribution of words in literary texts (Voloshynovska, 2011) was applied. The slope in rank–activity distribution of single cells, interpreted as differentiation tendency index (DTI), was suggested for heterogeneity quantitation. The DTI is independent of the population size, normalization procedures, and measure units, making its use general and universal.

In this study, the applicability of both developed indices, HC and DTI, was first tested on SIP–nanoSIMS datasets acquired for two different bacterial strains. The HC facilitates routine quantitation of heterogeneity for monoclonal populations following unimodality and can be easily calculated for any single-cell-resolved dataset with Supplementary Table S1 provided in the Supplementary Material. The DTI allows for recognition of subpopulations and expresses the heterogeneity showing multimodality. To show the wide applicability of the HC index and DTI, heterogeneity was also quantitated for datasets of flow cytometry and optical microscopy experiments. To our knowledge, this is the first time that such an approach was applied broadly for quantitation and comparison of phenotypic heterogeneity. Indeed, the two indices can be calculated for any numerically expressed feature at the single-cell level, since they are not bound to either any unit of measure or any particular technique.

## Materials and Methods

### Cultivation and Stable Isotope Labeling

*Pseudomonas putida* strain KT2440 (DSM 6125) and *Pseudomonas stutzeri* (environmentally isolated) were grown under aerobic conditions with mineral salts medium (MSM) containing (g/L) NH_4_Cl (0.3), KH_2_PO_4_ (0.2), NaCl (1), MgSO_4_ · 7H_2_O (0.5), and CaCl_2_ · 2H_2_O (0.1) supplemented with NaHCO_3_ (30 ml/L) as a buffer system. The medium was supplemented with 1 ml of a vitamin mix solution in phosphate buffer [10 mM NaH_2_PO_4_ · H_2_O, pH 7.1, containing, in milligrams per liter, 4-aminobenzoic acid, 40; d(+)-biotin, 10; nicotinic acid, 100; Ca-d(+)-pantothenate, 50; pyridoxine dihydrochloride, 150; folic acid, 40; and lipoic acid, 15] and with 1 ml of chelated trace element solution (containing, in milligrams per liter, Na_2_EDTA, 5,200; H_3_BO_3_, 10; MnCl_2_ · 4H_2_O, 5; FeSO_4_ · 7H_2_O, 2100; CoCl_2_ · 6H_2_O, 190; NiCl_2_ · 6H_2_O, 24; CuCl_2_ · 2H_2_O, 10; and ZnSO_4_ · 7H_2_O, 144; pH adjusted to 6.0 with 1 M NaOH). The vitamin mix and trace element solutions were prepared as previously described (Widdel, 2010). Both bacterial cultures were prepared by inoculating 1 ml of bacterial suspension in 29 ml of mineral medium in a sealed 140-ml serum bottle. The cultures were grown at 30°C with orbital shaking (100 rpm) with 5 mM acetate as growth substrate. Acetate is an intermediate of many cellular biosynthetic pathways, therefore a good candidate to track single-cell anabolic activity with the SIP–nanoSIMS approach. Stable isotope labeling was performed by adding [^13^C_2_]-acetate (Sigma Aldrich) to reach 20 at% of ^13^C relative to the total carbon in the growth substrate. A stock solution of the isotope-labeled acetate was prepared in MSM and filter-sterilized before use. Cultures were supplied with [^13^C_2_]-acetate at the onset of their exponential growth phase. Samples were collected at three different time points within the exponential growth phase and prepared for nanoSIMS analysis.

### Sample Preparation for NanoSIMS Analysis

Each sample was fixed overnight with 3% glutaraldehyde in sodium cacodylate buffer (0.2 M, pH 7.4, EM Science) at 4°C. Filters (0.22 μm pore size, 25 mm, GTTP type, Merck) were coated with a 30 nm gold–palladium layer and mounted inside a stainless steel syringe 25 mm filter holder (Sartorius). Bacterial cells were filtered; washed twice with cacodylate buffer; incubated with 1% H_2_O_2_ in cacodylate buffer, for 30 min; dehydrated via increasing concentrations of ethanol in water (30 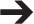
50 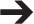
70 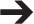
80 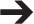
90 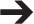
96 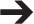
100); and dried upon 20 cycles in a critical point drying device (Leica EM CPD 300a, Germany).

### NanoSIMS Measurement

NanoSIMS analysis parameters were optimized in order to achieve the appropriate precision at the single-cell level [Supplementary Information (SI)]. From each filter of *P. putida* and *P. stutzeri*, a piece of 5 mm in diameter was cut and mounted on the 24-holes-holder for nanoSIMS measurement. Single-cell analysis was performed using a nanoSIMS-50L instrument (Cameca) acquiring the following seven molecular ion species (masses) ^16^O^−^, ^12^${\text{C}}_{2}^{-}$, ^13^C^12^C^−^, ^12^C^14^N^−^, ^13^C^14^N^−^, ^32^S^−^, and ^31^P^16^${\text{O}}_{2}^{-}$. The MRP was set above 7,000. Samples areas of 100 × 100 μm^2^ were pre-implanted with 100 pA of a 16 keV cesium (Cs^+^) primary ion beam for 5 min before measurements. Smaller areas within 20 × 20 μm^2^ field of view (FoV) were analyzed with a 4 pA primary Cs^+^ ion beam, a nominal size of 100 μm for the entrance slit, 40 μm exit slits, and an energy slit cutting 20% of secondary ion intensity at the high-energy distribution tail. Samples were scanned with a 512 × 512-pixel raster size and a dwell time of 2 ms/pixel. In total, 30 planes were acquired, ensuring complete sample consumption in each FoV analyzed; 11 of those were accumulated and corrected for lateral drifting using the Look@NanoSIMS (LANS) software (Polerecky et al., 2012). Regions of interest (RoI) were drawn manually around each single cell using the ^12^C^14^N^−^ map as a biomass distribution template supported by the cell image acquired in secondary electron signal. Isotope ratio data were exported and further processed with OriginPro 2019 software for statistical analysis and graphing. The relative assimilation (*K*_*A*_) values were calculated with the template provided in Stryhanyuk et al. (2018) and used for further numerical analysis and graphical representations.

### Cell Cultivation and Preparation for Flow Cytometric Analysis

After pre-cultivation on peptone medium (Medium 1, DSMZ), *P. putida* KT2440 was inoculated in Erlenmeyer flasks with 50 ml of M9 leucine medium and 1 g/L of acetate as carbon and energy source and grown at 30°C and 125 rpm. The strain grew with a μ_max_ of 0.62 h^−1^. After 24 h of incubation, acetate was added to the cultures again to reach a concentration of 1 g/l. Cell suspensions (1–3 ml) were sampled hourly and centrifuged for 10 min (3,200 × *g* at 4°C). The resulting pellets were resuspended in 4 ml of formaldehyde solution [1 ml of 8% (wt/vol) formaldehyde/phosphate-buffered saline (PBS) in 3 ml of PBS] and incubated at 4°C for 30 min. After a final centrifugation, the pellets were resuspended in 4 ml of 70% (vol/vol) ethanol/bi-distilled H_2_O and stored at −20°C. For flow cytometric analysis, the cells were stained according to Koch et al. (2013). In short, 1 ml of the fixed sample was centrifuged, and the pellet was resuspended in 2 ml of PBS (6 mM Na_2_HPO_4_, 1.8 mM NaH_2_PO_4_, and 145 mM NaCl with bi-distilled H_2_O, pH 7). The optical density (OD) of the well-mixed samples was measured (*d* = 0.5 cm; λ = 700 nm) and adjusted to 0.04 with PBS. After centrifugation of 1 ml of this solution for 10 min (3,200 × *g* at 4°C), the supernatant was discarded. The cell pellet was resuspended in 0.5 ml of permeabilization buffer (0.1 M citric acid, 4.1 mM Tween 20, and bi-distilled H_2_O) and incubated at room temperature for 20 min. After a further centrifugation step, the supernatant was discarded, and the cells were resuspended in 1 ml of DNA dye solution for overnight staining at room temperature until cytometric measurement [0.68 μM 4′,6-diamidino-2-phenylindole (DAPI), Sigma-Aldrich, in 417 mM Na_2_HPO_4_/NaH_2_PO_4_ buffer, 289 mM Na_2_HPO_4_, and 128 mM NaH_2_PO_4_ with bi-distilled H_2_O, pH 7].

### Flow Cytometric Analysis

The DAPI-stained *P. putida* KT2440 single cells were analyzed with a MoFlo Legacy cell sorter (Beckman-Coulter, Brea, California, USA) equipped with two lasers. The forward scatter (FSC) and side scatter (SSC) signals were measured using a blue laser (488 nm, 400 mW, Genesis MX488-500 STMOPS, Coherent, Santa Clara, California, USA). The FSC signal (band-pass filter 488 ± 5 nm, neutral density filter 1.9) is an optical characteristic related to cell size, whereas the SSC signal (trigger signal, band-pass filter 488 ± 5 nm, neutral density filter 1.9) is related to cell density. The DAPI fluorescence (band-pass filter 450 ± 32.5 nm) was excited by a UV laser (355 nm, 150 mW, Xcyte CY-355-150, Lumentum, Milpitas, California, USA). The resulting scatter and fluorescence signals were detected by photomultiplier tubes (Hamamatsu Photonics, models R928 and R3896; Hamamatsu City, Japan). The fluidic system was run at 56 psi (3.86 bar) with sample overpressure at a maximum of 0.3 psi (0.02 bar) and a 70 μm nozzle. The sheath fluid consisted of 10× sheath buffer (19 mM KH_2_PO_4_, 38 mM KCl, 166 mM Na_2_HPO_4_, and 1.39 M NaCl with bi-distilled H_2_O) that was diluted to a 0.2× working solution with 0.1 μm filtrated bi-distilled H_2_O. The instrument was calibrated in the linear and logarithmic ranges prior to all measurements. Blue fluorescent 1 μm beads [FluoSpheres F8815 (350/440), lot no.: 69A1-1] and 2 μm yellow-green fluorescent beads [FluoSpheres F8827 (505/515), lot no.: 1717426, both from Molecular Probes, Eugene, Oregon, USA] were used for linear calibration. Blue fluorescent 0.5 and 1 μm beads [both Fluoresbrite BB carboxylate microspheres (360/407), lot nos.: 552744 and 499344, PolyScience, Niles, Illinois, USA] and red fluorescent 1 μm beads [FluoSpheres F8816 (625/645), lot no.: 24005W] were used for calibration in the logarithmic scale and added to every sample to secure instrument stability. For every sample, ~10^5^ cells were analyzed. Cell gate definition is shown in Supplementary Figure S11. The analog current signal delivered by the photomultiplier tubes was amplified and converted into a voltage signal within a range of 0–10 V in a preamplifier. This voltage signal was then amplified onto a 4-decade logarithmic range. The analog-to-digital converter assigns each event in this signal to one of 1,024 intensity channels according to the event peak height.

## Results and Discussion

In the present study, the heterogeneity quantitation approach was developed with considerations of single-cell anabolic activity derived from nanoSIMS data. The applicability of the suggested heterogeneity indices was proved for the analysis of (i) cellular DNA content measured with flow cytometry of DAPI-stained single cells and (ii) length of single cells acquired with fluorescence microscopy.

### Quantitation of Single-Cell Anabolic Activity and Evaluation of Activity Distribution

The two *Pseudomonas* strains were grown in batch cultures with defined starting concentrations of ^13^C-labeled and unlabeled acetate. Afterwards, SIP–nanoSIMS experiments were performed to follow the biosynthetic (anabolic) activity and to compare the metabolic differences within the two monoclonal populations. Single-cell activity was expressed as relative assimilation (*K*_*A*_, Equation 1) (Stryhanyuk et al., 2018):

(1) $$\begin{array}{l} K_{A}=\frac{R_{f}-R_{i}}{\left(R_{i}+1\right)\times \left(D_{gs}\times \left(R_{f}+1\right)-R_{f}\right)} \end{array}$$

where *R*_*f*_ and *R*_*i*_ are the final and initial cellular isotope ratios, respectively, and *D*_*gs*_ is the fraction of heavy isotope in the growth substrate during incubation.

The distribution of single cells in *K*_*A*_ was considered for the analysis of anabolic heterogeneity within each population. The applicability of existing dispersion expressions for quantitation of heterogeneity is limited by the shape of the distribution. When in a certain population single cells fit a normal distribution in their anabolic activity, the dispersion in *K*_*A*_*i*__ (*i* ∈ [1, *n*]) values around the mean (${\bar{K}}_{A}$),

(2) $$\begin{array}{l} \text{mean}\left({K_{A}}_{i}\right)\equiv {\bar{K}}_{A}=\frac{\sum_{i=1}^{n}{K_{A}}_{i}}{n}, \end{array}$$

is expressed for a population of *n* cells as standard deviation (σ):

(3) $$\begin{array}{l} \sigma =\sqrt{\frac{\sum_{i=1}^{n}{\left({K_{A}}_{i}-\;{\bar{K}}_{A}\right)}^{2}}{n}}. \end{array}$$

The 2σ range represents the distribution width (DW) comprising 68.2% of the population distributed equally (±34.1%) around $\;{\bar{K}}_{A}$. The probability density function of a normal distribution reveals the symmetrical bell-shaped profile with the probability maximum (*mode*) and the median centered at the mean value (${\bar{K}}_{A}$).

The variability inside the population can be thus quantified by the CV calculated as

(4) $$\begin{array}{l} \text{CV}=\frac{\sigma }{{\bar{K}}_{A}}. \end{array}$$

In previous studies, the CV has been applied for the evaluation of population heterogeneity and biological noise (Grover and Woldringh, 2001; Bar-Even et al., 2006; Newman et al., 2006; Simpson et al., 2009; Schreiber et al., 2016; Nikolic et al., 2017). However, the biosynthetic activity of individual cells may deviate from a normal distribution even in monoclonal populations, which calls for more comprehensive approaches to evaluate heterogeneity at the single-cell level.

#### Quantitation of the Distribution Width for Heterogeneity Measurement

**Figure 1:**
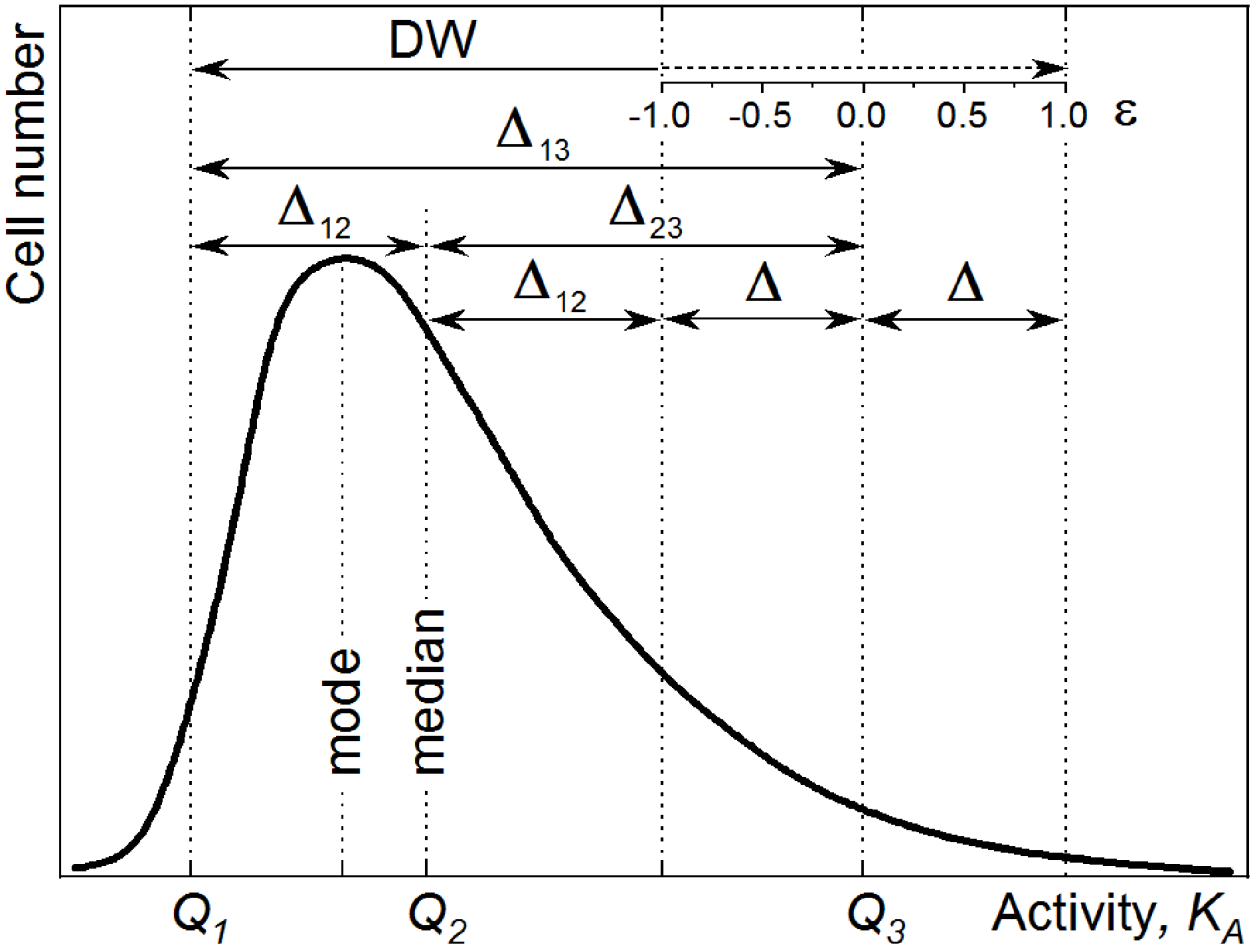
Representative sketch for the derivation of distribution width (DW) in the case of skewed distribution.

Even in actively growing populations, the single-cell anabolic activity distribution cannot always be described with a symmetric probability density function. When a distribution of single cells in anabolic activity is asymmetric, the ${\bar{K}}_{A}$ is displaced from the distribution maximum (*mode*, Figure 1), rendering the CV (Equation 4) unsuitable. The median value

(5) $$\begin{array}{l} {\tilde{K}}_{A}=\text{median}\left({K_{A}}_{i}\right),\;i\in \left[1,n\right], \end{array}$$

is more appropriate since it represents exactly the centroid of an asymmetric distribution rather than the average of its terms (like ${\bar{K}}_{A}$ does). Thus, to express a robust measure of heterogeneity, considering the distribution asymmetry, the centroid of the activity distribution derived as ${\tilde{K}}_{A}$ has to substitute the ${\bar{K}}_{A}$ in the denominator of Equation (4). Additionally, to keep the quantitation of heterogeneity consistent with the CV, half of the distribution width (i.e., DW/2) has to be considered in the nominator, exactly as half of the 2σ range is considered in the nominator of the CV expression (Equation 4). With the fulfillment of these two objectives, that is, robustness and consistency, the HC has been developed. By means of this index, the anabolic heterogeneity of a population can be expressed with the DW in *K*_*A*_ normalized to the ${\tilde{K}}_{A}$ as follows:

(6) $$\begin{array}{l} \text{HC}=\frac{1}{2}\times \frac{\text{DW}}{{\tilde{K}}_{A}}. \end{array}$$

The DW can be measured in different ways, for example, as standard deviation (σ); median absolute deviation (MAD); and min–max, interquantile, or interquartile range. With the MAD, the deviation of a single-cell *K*_*A*_*i*__ value from the median centroid (${\tilde{K}}_{A}$) of activity distribution is expressed as

(7) $$\begin{array}{l} \text{MAD}=\text{median}\left(\left|{K_{A}}_{i}-{\tilde{K}}_{A}\right|\right)\;,\;i=1,\ldots ,n. \end{array}$$

In the case of normal distribution, the 2 × MAD range (MAD ≈ 0.67449 × σ) comprises 46% of the population equally distributed (±23%) around ${\tilde{K}}_{A}$. With the DW measured as 2 × MAD, the HC expression (Equation 6) returns the COD (Bonett and Seier, 2006):

(8) $$\begin{array}{l} \text{COD}=\frac{\text{MAD}}{{\tilde{K}}_{A}}. \end{array}$$

Thus, as a measure of heterogeneity, COD substantially reduces sensitivity to the distribution asymmetry.

Expressing the DW in terms of quantiles *Q*_(*P*)_ provides the possibility of tuning the sensitivity of a heterogeneity index to the distribution asymmetry. The *Q*_(*P*)_ quantile splits the distribution into two parts comprising *P*% and 100 – *P*% of a population. A *Q*_(*P*)_ quantile equals the *K*_*A*_ value, which is higher than those values revealed by *P*% of cells in the population. The percentile *P* (%) varies within a range of 0–100%, where 100% corresponds to the whole population. Thus, quantile *Q*_(50)_ of *K*_*A*_ distribution equals the median value ${\tilde{K}}_{A}$. With a set of three quantiles (*Q*_1_, *Q*_2_, and *Q*_3_; Figure 1),

$$\begin{array}{l} Q_{1}\equiv Q_{\left(P\right)};\;Q_{2}\equiv Q_{\left(50\right)};\;Q_{3}=Q_{\left(100-P\right)}, \end{array}$$

the DW can be expressed as an interquantile range: Δ_*i*−*j*_ = *Q*_*j*_ – *Q*_*i*_.

When the percentile *P* equals 25%, the quantiles *Q*_1_, *Q*_2_, and *Q*_3_ become *Q*_(25)_, *Q*_(50)_, and *Q*_(75)_, respectively, and are called quartiles dividing a cell population into four quarters with an equal cell number. The following measures of DW are expressed in terms of quartiles:

interquartile range, Δ_1−3_ = *Q*_(75)_ – *Q*_(25)_;

semi-interquartile deviation, *Q*_*d*_ = 0.5 × (*Q*_(75)_ – *Q*_(25)_); and

CQD = (*Q*_(75)_ – *Q*_(25)_)/(*Q*_(75)_ + *Q*_(25)_).

The expression of DW in quartiles provides a reduced sensitivity to the distribution asymmetry, since it considers just 50% of the population within the interquartile range. To tune the asymmetry contribution into a heterogeneity index, one can expand or reduce the interquantile range by varying the percentile *P*-value. Hence, the maximum sensitivity is achieved with DW expressed with *Q*_(0)_ and *Q*_(100)_ as the [min–max] range. The calculation of heterogeneity indices with different definitions of DW is implemented in Supplementary Table S1. However, to render the HC (Equation 6) consistent with the CV (Equation 4), the interquantile range Δ_1−3_ = *Q*_(84)_ – *Q*_(16)_ was considered as DW in Equation (6) for further steps of the heterogeneity quantitation. In the case of normal distribution, the HC value

(9) $$\begin{array}{l} \text{HC}=\frac{1}{2}\times \frac{Q_{\left(84\right)}-Q_{\left(16\right)}}{Q_{\left(50\right)}}=\frac{\Delta_{1-3}}{2\times {\tilde{K}}_{A}} \end{array}$$

equals approximately the CV (Equation 4). Indeed, with a limited number of single cells, quantiles are defined by real single-cell values, whereas the calculated mean value does not necessarily match any experimentally observed one (because it represents the average of the observed values). For a discrete distribution, the quantiles *Q*_(16)_, *Q*_(50)_, and *Q*_(84)_ return single-cell activity values that are not matching exactly the mean and the borders of the 2σ range (i.e., the *Q*_(16)_ and *Q*_(84)_ values are approximately matching the ±34.1% points around the mean); therefore, CV and HC values cannot be exactly the same.

#### HC for Skewed Distributions

The heterogeneity in the anabolic activity of monoclonal population causes the asymmetry of the *K*_*A*_ distribution profile (Figure 1). For this reason, an asymmetry measure was considered in the expression of anabolic heterogeneity. The asymmetry is revealed as a distribution skew toward higher values (positive skew) or lower values (negative skew). The skew causes the displacement of the centroid (*Q*_2_) from the maximum (*mode*), gaining the difference Δ = Δ_2−3_ – Δ_1−2_ between the interquantile distances (Figure 1). The degree of distribution asymmetry is measured with skewness (*Sk*) expressed in interquantile distances as

(10) $$\begin{array}{l} Sk=\frac{\left(\Delta_{2-3}-\Delta_{1-2}\right)}{\Delta_{1-3}}\;. \end{array}$$

According to the interquantile range considered in the nominator of Equation (9), the difference Δ is equal to

(11) $$\begin{array}{l} \Delta =\Delta_{2-3}-\Delta_{1-2\;}=\left(Q_{\left(84\right)}-Q_{\left(50\right)}\right)-\left(Q_{\left(50\right)}-Q_{\left(16\right)}\right) \end{array}$$

Taking Equation (10) into account, Δ can be expressed in terms of *Sk* and Δ_1−3_ as follows:

(12) $$\begin{array}{l} \Delta =|\Delta_{2-3}-\Delta_{1-2}|=|Sk|\times \Delta_{1-3} \end{array}$$

To operate with positive Δ values and thus make Equation (12) applicable for positively and negatively skewed distributions, the absolute value of skewness |*Sk*| was considered.

With the difference Δ (Equation 12), the skewness was taken into account for the quantitation of heterogeneity. Expression of DW as the sum of the interquantile range Δ_1−3_ and the Δ value allows for the enhancement of the HC sensitivity to the data points scattered over the distribution tails (Figure 1).

(13) $$\begin{array}{l} \;DW=\Delta_{1-3}+\Delta \\ \Delta_{1-3}=Q_{\left(84\right)}-Q_{\left(16\right)} \end{array}$$

The Δ expression in Equation (12) allows for rewriting of the DW (Equation 13) in terms of *Sk* and Δ_1−3_ as follows:

(14) $$\begin{array}{l} \text{DW}=\Delta_{1-3}+|Sk|\times \Delta_{1-3}=\left(1+|Sk|\right)\times \Delta_{1-3} \end{array}$$

With the DW expressed in terms of skewness and interquantile distance (Equation 14), the HC expression (Equation 6) can be now rewritten as

(15) $$\begin{array}{l} \text{HC}=\frac{1}{2}\times \frac{DW}{{\tilde{K}}_{A}}=\frac{\left(1+|Sk|\right)\times \Delta_{1-3}}{2\times Med} \end{array}$$

or

(15′) $$\begin{array}{l} \text{HC}=\frac{\left(1+|Sk|\right)\times \left(Q_{3}-Q_{1}\right)}{2\times Q_{2}}. \end{array}$$

The introduction of the *Sk* weighting factor ε into Equations (15 and 15′) as

(16) $$\begin{array}{l} \text{HC}=\frac{\left(1+\varepsilon \times |Sk|\right)\times \Delta_{1-3}}{2\times Q_{2}}=\frac{\left(1+\varepsilon \times |Sk|\right)\times \left(Q_{3}-Q_{1}\right)}{2\times Q_{2}} \end{array}$$

provides the possibility of adjusting the HC sensitivity to the skew by varying the DW within the Δ_1−3_ ± Δ range with ε ∈ [−1; 1]. With ε = 1, the DW = Δ_1−3_ + Δ (Figure 1) and Equation (16) turns to Equation (15). By setting the weighting factor as ε = −1 (Figure 1), we can narrow down the DW to Δ_1−3_ – Δ, and the sensitivity is reduced. With ε = 0, the term ε × |*Sk*| is nullified, and Equation (16) turns to Equation (9); even in this case though, it has to be considered that the skewness affects the HC value since Δ_1−3_ and ${\tilde{K}}_{A}$ values are intrinsically influenced by the distribution skew.

If the *K*_*A*_ distribution is normal (ε × |*Sk*| = 0, $Q_{2}={\bar{K}}_{A}\;$, Δ_1−3_ = *Q*_3_ – Q_2_ = 2σ), the HC expressed with Equation (16) becomes approximately equal to CV (Equation 4).

#### Correction of the HC for the CSE

In the calculation of anabolic activity (*K*_*A*_) from SIP–nanoSIMS data, the changes in cellular isotopic composition upon incubation with stable isotope labels are considered at the single-cell level (Stryhanyuk et al., 2018). During nanoSIMS analysis, the quantification of cellular isotope composition is based on the isotope ion counts accumulated in imaging mode for each pixel within a single cell. Therefore, it is important to evaluate the effect of CSE of the acquired data on the derived *K*_*A*_ values considered for quantitation of heterogeneity.

The CSE is derived as the square-root of secondary ion counts (*N*) (Sprawls, 1995).

$$\begin{array}{l} \text{CSE}=\sqrt{N} \end{array}$$

Taking as example Carbon (C) isotopes, the calculation of their ratio (*R*) considers the counts of ^13^C^−^ and ^12^C^−^ isotope ions as

$$\begin{array}{l} R=\frac{N_{13_{C}}}{N_{12_{C}}} \end{array}$$

and implies the propagation of CSE (Fitzsimons et al., 2000) into the Counting Statistics Variations of isotope ratios (*CSV*_*R*_).

(17) $$\begin{array}{l} {\text{CSV}}_{R}=\sqrt{{\left(\frac{\partial R}{\partial N_{13_{C}}}\times {\text{CSE}}_{13_{C}}\right)}^{2}+{\left(\frac{\partial R}{\partial N_{12_{C}}}\times {\text{CSE}}_{12_{C}}\right)}^{2}}=\\ \;=\sqrt{{\left(\frac{{\text{CSE}}_{13_{C}}}{N_{12_{C}}}\right)}^{2}+{\left(\frac{N_{13_{C}}\times {\text{CSE}}_{12_{C}}}{N_{12_{C}{\;}^{2}}}\right)}^{2}} \end{array}$$

Thus, the presence of the isotope ratios (*R*_*i*_ and *R*_*f*_) in the expression of *K*_*A*_ (Equation 1) implicates the CSE propagation (Fitzsimons et al., 2000) into the variation of calculated *K*_*A*_ values (*CSV*_*K*_*A*__).

(18) $$\begin{array}{l} \text{CS}{\text{V}}_{K_{A}}=\sqrt{{\left(\frac{\partial K_{A}}{\partial R_{f}}\times \text{CS}{\text{V}}_{R_{f}}\right)}^{2}+{\left(\frac{\partial K_{A}}{\partial R_{i}}\times \text{CS}{\text{V}}_{R_{i}}\right)}^{2}}=\\ \begin{array}{ll} \;= & \sqrt{\begin{array}{c} {\left(\frac{(R_{f}+1)\times CSV_{R_{i}}}{\;{\left(R_{i}+1\right)}^{2}\times (R_{f}-D_{gs}\times (R_{f}+1))}\right)}^{2}\\ +{\left(\frac{(D_{gs}\times (R_{i}+1)-R_{i})\times \text{CS}{\text{V}}_{R_{f}}}{\;(R_{i}+1)\times {\left(R_{f}-D_{gs}\times (R_{f}+1)\right)}^{2}}\right)}^{2} \end{array}} \end{array} \end{array}$$

Consequently, the propagation of CSE introduces the term of Counting Statistics Heterogeneity (*CSH*_*K*_*A*__) as an error into the anabolic activity derived from the SIP–nanoSIMS experiment that can be expressed as:

(19′) $$\begin{array}{l} {\text{CSH}}_{K_{A}}=\frac{{\text{CSV}}_{K_{A}}}{{\tilde{K}}_{A}} \end{array}$$

The correction for the CSE propagation is especially necessary when the HC value (Equation 16), derived for the cell anabolism (*K*_*A*_), approaches the *CSH*_*K*_*A*__ term (Equation 19).

**Figure 2:**
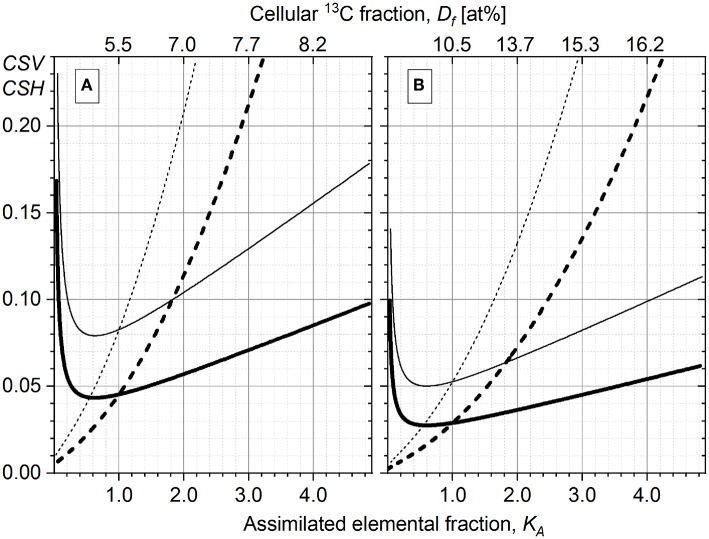
Counting statistics variation *CSV*_*K*_*A*__ (Equation 18, dashed line) and counting statistics heterogeneity *CSH*_*K*_*A*__ (Equation 19, solid line) calculated for the ^13^C isotope fraction *D*_*gs*_ of the growth substrate corresponding to 10 at% **(A)** and 20 at% **(B)**. Two values of the total carbon ion counts are represented: *N* = 15 × 10^3^ (thin lines) and *N* = 5 × 10^4^ (thick lines). The initial isotope ratio *R*_*i*_ = 0.011, corresponding to 1% of ^13^C cellular abundance, and the *CSV*_*R*_*i*__ = 0 are considered.

The *CSV*_*K*_*A*__ and the corresponding *CSH*_*K*_*A*__ depend on *K*_*A*_ (Figure 2) and are defined with

- accumulated ion counts (*N*) for both isotopes,
- isotope-labeled substrate content (*D*_*gs*_) in the growth substrate.

Considering different *CSV*_*K*_*A*__ values for *Q*_*i*_ quantiles of a certain activity distribution, the CSE propagation into the HC can be expressed more precisely with *HC* in the following way:

(19) $$\begin{array}{l} \Delta \text{HC}=\sqrt{\overset{3}{\underset{i=1}{\sum }}{\left[\frac{\partial \text{HC}}{\partial Q_{i}}\times CSV\left\{Q_{i}\right\}\right]}^{2}} \end{array}$$

where *CSV*{*Q*_*i*_} are the *CSV*_*K*_*A*__ values (Equation 18) corresponding to *Q*_*i*_ quantiles (*i* = 1, 2, 3) in an experimentally derived distribution of cellular anabolic activity. With the denotation of constant term

$$\begin{array}{l} \frac{\left(1+\varepsilon \times |Sk|\right)}{2}\equiv C \end{array}$$

in the HC expression (Equation 16), the partial derivatives are calculated as follows:

$$\begin{array}{r} \frac{\partial \text{HC}}{\partial {\text{Q}}_{1}}=-\frac{\text{C}}{{\text{Q}}_{2}};\\ \frac{\partial \text{HC}}{\partial {\text{Q}}_{2}}=-\frac{\text{C}\times ({\text{Q}}_{3}-{\text{Q}}_{1})}{{\left({\text{Q}}_{2}\right)}^{2}};\\ \frac{\partial \text{HC}}{\partial {\text{Q}}_{3}}=\frac{\text{C}}{{\text{Q}}_{2}}. \end{array}$$

The correction of HC for the CSE propagation was implemented via the subtraction of the *CSV*_*K*_*A*__ from the dispersion of experimental data in the following steps:

1. calculation of the *CSV*_*K*_*A*__ for *K*_*A*_ values corresponding to *Q*_1_ and *Q*_3_ quantiles (i.e., *CSV*{*Q*_1_} and *CSV*{*Q*_3_}) with Equation (18) accounting for the isotope ion counts ($N_{12_{C}}$ and $N_{13_{C}}$) and the fraction of labeling isotope (*D*_*gs*_) in the growth substrate;
2. calculation of the corrected interquantile range Δ_1−3_*corr*__ by subtracting the *CSV* defined by *CSV*{*Q*_1_} and *CSV*{*Q*_3_} (derived in the previous step)
  $$\begin{array}{l} \Delta \text{CSV}=\sqrt{{\left(\text{CSV}\left\{Q_{1}\right\}\right)}^{2}+{\left(\text{CSV}\left\{Q_{3}\right\}\right)}^{2}} \end{array}$$
  from the interquantile range Δ_1−3_ = *Q*_3_−*Q*_1_ in the following way:
  (20) $$\begin{array}{l} \Delta_{1-3_{{\;}_{corr}}}=\sqrt{{\left(Q_{3}-Q_{1}\right)}^{2}-[{\left(CSV\{Q_{1}\}\right)}^{2}+{\left(CSV\{Q_{3}\}\right)}^{2}]} \end{array}$$
3. calculation of the *HC*_*corr*_ with Equation (16) substituting the Δ_1−3_ with the Δ_1−3_*corr*__ in the numerator and considering the experimental median $Q_{2}={\tilde{K}}_{A}$ in the denominator:
  (21) $$\begin{array}{l} {\text{HC}}_{\text{corr}}=\frac{\left(1+\varepsilon \times |Sk|\right)\times {\Delta_{1-3}}_{corr}}{2\times {\tilde{K}}_{A}}\;. \end{array}$$

The HC_*corr*_ error due to the CSE propagation into the HC_*corr*_ calculation can be expressed in the following way:

(21′) $$\begin{array}{l} \Delta {\text{HC}}_{\text{corr}}=\sqrt{\overset{3}{\underset{i=1}{\sum }}{\left[\frac{\partial HC_{corr}}{\partial Q_{i}}\times CSV\left\{Q_{i}\right\}\right]}^{2}}. \end{array}$$

$$\begin{array}{lll} \frac{\partial {\text{HC}}_{\text{corr}}}{\partial {\text{Q}}_{1}} & = & \frac{\text{C}}{{\text{Q}}_{2}}\times \frac{\left({\text{Q}}_{1}-{\text{Q}}_{3}\right)}{\sqrt{{\left({\text{Q}}_{3}-{\text{Q}}_{1}\right)}^{2}-\left[{\left(\text{CSV}\left\{{\text{Q}}_{1}\right\}\right)}^{2}+{\left(\text{CSV}\left\{{\text{Q}}_{3}\right\}\right)}^{2}\right]}}\\ \frac{\partial {\text{HC}}_{\text{corr}}}{\partial {\text{Q}}_{2}} & = & -\frac{\text{C}}{{\left({\text{Q}}_{2}\right)}^{2}}\times \sqrt{{\left({\text{Q}}_{3}-{\text{Q}}_{1}\right)}^{2}-\left[{\left(\text{CSV}\left\{{\text{Q}}_{1}\right\}\right)}^{2}+{\left(\text{CSV}\left\{{\text{Q}}_{3}\right\}\right)}^{2}\right]}\\ \frac{\partial {\text{HC}}_{\text{corr}}}{\partial {\text{Q}}_{3}} & = & \frac{\text{C}}{{\text{Q}}_{2}}\times \frac{\left({\text{Q}}_{3}-{\text{Q}}_{1}\right)}{\sqrt{{\left({\text{Q}}_{3}-{\text{Q}}_{1}\right)}^{2}-\left[{\left(\text{CSV}\left\{{\text{Q}}_{1}\right\}\right)}^{2}+{\left(\text{CSV}\left\{{\text{Q}}_{3}\right\}\right)}^{2}\right]}} \end{array}$$

Figure 2 shows the comparison of *CSH*_*K*_*A*__ (solid lines) and *CSV*_*K*_*A*__ (dashed lines) for different experimental conditions (acquired ion counts *N* and concentrations of isotope-labeled growth substrate *D*_*gs*_). The *CSH*_*K*_*A*__ and the *CSV*_*K*_*A*__ terms are enhanced with lower ion counts (*N* = 1.5 × 10^4^, thin lines) and lower *D*_*gs*_ (10 at%, Figure 2A) as compared with the case when higher *N* and *D*_*gs*_ values are considered, that is, *N* = 5 × 10^4^ (thick lines) and *D*_*gs*_ = 20 at% (Figure 2B). For *K*_*A*_ > 0.5, the steepness of the *CSV*_*K*_*A*__ and *CSH*_*K*_*A*__ profiles increases with the reduction of *N* and *D*_*gs*_.

High *CSH*_*K*_*A*__ values at *K*_*A*_ < 0.5 are due to the division of *CSV*_*K*_*A*__ by a small ${\tilde{K}}_{A}$ value (Equation 19). With the *K*_*A*_ approaching zero, the *CSV*_*K*_*A*__ becomes comparable with the DW and the *CSH*_*K*_*A*__ reaches its asymptote. These trends cause a huge error in HC calculation rendering the correction for CSE impossible and the HC value unreliable, when small changes in cellular heavy-isotope enrichment (small *R*_*f*_ − *R*_*i*_ difference in Equation 1; *K*_*A*_ → 0) are considered. Consequently, the anabolic heterogeneity cannot be expressed in terms of HC when cellular isotope content is close to its natural abundance (e.g., short-time incubation or time prior incubation with isotope-labeled substrates). Therefore, in this study HC values were not provided for samples before incubation with ^13^C-labeled acetate (hereafter T0).

The increase in *CSV*_*K*_*A*__ and *CSH*_*K*_*A*__ is steeper when the cellular isotope-label content (*D*_*f*_) approaches the *D*_*gs*_ value,

(22) $$\begin{array}{l} D_{f}=\frac{R_{f}}{\left(R_{f}+1\right)}. \end{array}$$

In biological systems, the cellular isotope enrichment *D*_*f*_ may approach but not exceed the *D*_*gs*_ because the cells retain the original unlabeled material along cell division. The only exception is possible when metabolic reactions reveal an isotopic fractionation factor smaller than the unit ($\frac{R_{gs}}{R_{f}}=\alpha <1$) (Stryhanyuk et al., 2018). The values of *K*_*A*_, *CSV*_*K*_*A*__, and *CSH*_*K*_*A*__ show a strong increase at higher *D*_*f*_ especially when approaching their asymptote at *D*_*f*_ = *D*_*gs*_. It is therefore not recommended to quantify the anabolic activity and the anabolic heterogeneity when the cellular isotope-label content *D*_*f*_ exceeds 0.6 × *D*_*gs*_ (Stryhanyuk et al., 2018). An increase of *D*_*f*_ requires high amount of labeled substrate to be assimilated when *D*_*f*_ approaches the asymptote at *D*_*gs*_. The *D*_*f*_ proximity to the corresponding *K*_*A*_ asymptote causes a strong increase in $\frac{\partial K_{A}}{\partial R_{f}}$ Equation (18) providing a higher error in *K*_*A*_ calculation as well as an enhancement of the CSE propagation into the *CSV*_*K*_*A*__. In turn, the increase in *CSV*_*K*_*A*__ intensifies the *CSH*_*K*_*A*__ term (Equation 19) of heterogeneity inaccuracy originated from the CSE.

#### HC Applicability to NanoSIMS Data

To test the applicability of the derived HC (Equation 16) on quantitation of anabolic heterogeneity, two *Pseudomonas* strains were incubated with ^13^C-labeled acetate, sampled at different time points during their exponential growth and analyzed by nanoSIMS. The acquired isotope distribution maps were used to calculate the single-cell isotope content *D*_*f*_.

**Figure 3:**
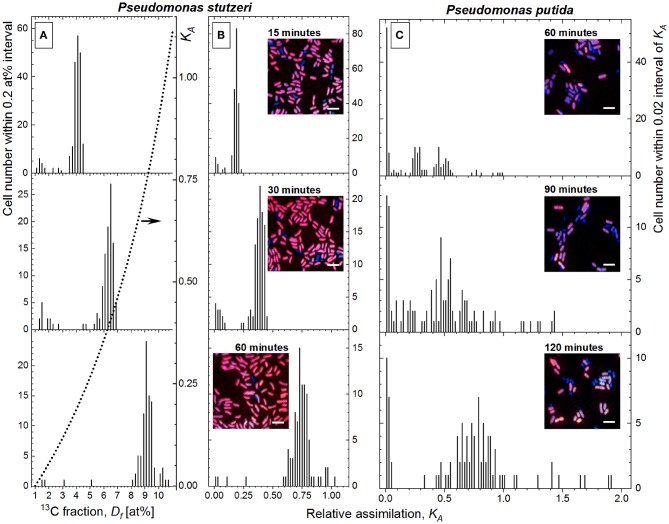
The histograms of *Pseudomonas stutzeri* cell distribution in their ^13^C fraction (*D*_*f*_, **A**) and their relative assimilation *K*_*A*_ **(B)** plotted at three different time points of incubation with ^13^C-labeled acetate. The dependence of *K*_*A*_ on *D*_*f*_ is shown in **(A)** with the dotted line and right *Y*-axis-*K*_*A*_. **(C)** Distribution of *Pseudomonas putida* cells in relative assimilation *K*_*A*_. The RGB insets show the overlay of ^13^C^14^N^−^/^12^C^14^N^−^ (red), ^31^P^16^${\text{O}}_{2}^{-}$ (green), and ^12^C^14^N^−^ (blue) acquired with nanoscale secondary ion mass spectrometry (nanoSIMS) at different time points for both bacterial strains.

The dependence of *K*_*A*_ on *D*_*f*_ (Equations 1 and 22) is not linear (dotted line in Figure 3A, right *Y*-axis); for example, to increase the ^13^C content (*D*_*f*_) from 7 to 10 at%, the cells have to assimilate twice (×2.08) more carbon (higher steepness causing larger *K*_*A*_ interval), as compared with the same (3 at%) increase in *D*_*f*_ from 2 to 5 at% (lower steepness causing smaller *K*_*A*_ interval). To illustrate this, the distribution of *P. stutzeri* cells in their ^13^C-fraction and *K*_*A*_ (Figures 3A,B) were shown. Due to this non-linearity, the dispersion of cells (DW) in *K*_*A*_ scale (Figure 3B) does not reproduce the one in *D*_*f*_ scale (Figure 3A) although the same *P. stutzeri* cells are represented in both *D*_*f*_ and *K*_*A*_ plots (Figures 3A,B). In comparison with the *D*_*f*_ distribution, the *K*_*A*_ distribution appears more compressed (Figures 3A,B) in the range of small *K*_*A*_ values (Figure 3A, 15 min); instead it is more stretched and better reveals the intra-population metabolic diversifications between cells with higher *K*_*A*_ values (Figure 3A, 60 min).

To compare the heterogeneity in anabolic activity of the two strains, the *K*_*A*_ distribution of *P. putida* cells (Figure 3C) were plotted with the same integration interval (0.02) as the one used for *P. stutzeri* histogram plots (Figure 3B). The histograms display different distribution in cellular anabolic activity between the two bacterial populations; however, this representation does not provide any quantitative measure of the heterogeneity in single-cell activity. For this reason, the developed HC-computation approach was applied for quantitation of heterogeneity and used afterwards for comparison between incubation time points of both strains.

As one would expect from an actively growing culture, the single-cell *K*_*A*_ values increase over the time of incubation with the isotope-labeled substrate. Meanwhile, the diversity in cellular anabolic activity causes the broadening of *K*_*A*_ distribution; that is, the DW varies over time. An increase in DW does not necessary indicate any rise of heterogeneity; namely, the HC value (Equation 15) is preserved when DW increases or decreases proportionally to the change in ${\tilde{K}}_{A}$ centroid. The scattering of single-cell anabolic activity (*K*_*A*_) revealed as DW increase and skew of *K*_*A*_ distribution, is indeed induced by the diversification in single-cell anabolism; that is, each cell incorporates a different amount of substrate at that specific time point.

The DW values were derived from the experimental data according to Equation (14). To account for the CSE, the corrected interquantile range (Δ_1−3_*corr*__, Equation 20) was considered in Equation (14) (instead of Δ_1−3_). The CSE does depend neither on the skew nor on the width of the distribution, but is defined by the *CSV*_*K*_*A*__ (Equation 18) depending on *K*_*A*_ term, collected isotope-ion counts ($N_{12_{C}}$ and$N_{13_{C}}$, Equation 17) and content of heavy isotope (*D*_*gs*_) in the growth substrate (Figure 2, dashed lines). Therefore, to account for CSE, the *CSV*_*K*_*A*__ values (*CSV*{*Q*_1_} and *CSV*{*Q*_3_}) were subtracted from the Δ_1−3_ interquantile range (Equation 20) without taking the skewness into account. The *CSV*_*K*_*A*__ dependence on *K*_*A*_ corresponding to the nanoSIMS applied experimental conditions for both *Pseudomonas* strains ($N=N_{12_{C}}+N_{13_{C}}=15\times 10^{3}$ counts, *D*_*gs*_ = 20 at%) is shown in Figure 2B with the thin dashed line; thin solid line (Figure 2B) represents the corresponding *CSH*_*K*_*A*__ dependence. For the CSE correction, the *CSV*_*K*_*A*__ values (Equation 18) were derived from experimental *K*_*A*_ values corresponding to *Q*_1_ and *Q*_3_ quantiles (i.e., *CSV*{*Q*_1_} and *CSV*{*Q*_3_}) in the activity distributions of both strains at every specific time point. The CSE-related error ΔHC (Table 1) of the uncorrected HC (Equation 16) can be expressed with Equation (19′). Considering the Δ_1−3_*corr*__ values, the HC_*corr*_ and HC_*corr*_ (Equations 21 and 21′) were calculated with ε = 0 to demonstrate numerically the effect of CSE correction in the resulted heterogeneity index *HC*_*corr*_ (Table 1).

**Table 1:** Effect of counting statistics error (CSE) correction on the heterogeneity coefficient (HC).

**Incubation time (min)**	**${\tilde{k}}_{A}$**	**HC ± ΔHC**		**Δ_1−3_**	***CSV*_*K*_*A*__**	**HC**_******corr******_ **± ΔHC**_******corr******_		CV^*^
		**HC**	**ΔHC**		***CSV*{*Q*_1_} *CSV*{*Q*_3_}**	**HC_corr_**	**ΔHC_corr_**	
***Pseudomonas putida***								
60	0.2451	0.9822	0.0878	0.4815	0.0042 0.0322	0.9799	0.0879	0.9728
90	0.4627	0.7434	0.0663	0.6882	0.0046 0.0497	0.7417	0.0663	0.7953
120	0.7291	0.6119	0.0524	0.8923	0.0042 0.0612	0.6104	0.0524	0.6234
***Pseudomonas stutzeri***								
15	0.1847	0.1377	0.0462	0.0509	0.0105 0.0131	0.1301	0.0487	0.3375
30	0.3837	0.1530	0.0367	0.1174	0.0158 0.0225	0.1488	0.0376	0.3437
60	0.7403	0.0886	0.0364	0.1312	0.0313 0.0434	0.0809	0.0398	0.2119

* *Coefficient of variation (CV) values were added for comparison with HC_corr_ value proposed in the present study*.

Despite small *CSV*_*K*_*A*__ values (0.01–0.04 for ${\tilde{K}}_{A}\in$ [0.15, 0.80]) throughout constant applied nanoSIMS experimental conditions, the CSE correction results in HC_*corr*_ deviating from the uncorrected HC in different extent for the analyzed strains (Table 1). The effect of CSE correction is negligible (0.23–0.25%) for *P. putida* whereas it becomes considerable (2.75–8.70%) for *P. stutzeri* strain. Such a difference in the correction effect can be explained by the DW expressed with Δ_1−3_*corr*__ in the nominator of HC_*corr*_ (Equation 21). The difference between HC and HC_*corr*_ is large when *CSV*_*K*_*A*__ (i.e., *CSV*{*Q*_1_} and *CSV*{*Q*_3_} values) are subtracted with Equation (20) from a relatively small Δ_1−3_ interquantile range, as observed for *P. stutzeri* (Figure 3B; Table 1). Instead *P. putida* revealed strong dispersion (large Δ_1−3_) of single-cell anabolic activity (Figure 3C; Table 1) values exceeding considerably the *CSV*_*K*_*A*__, thus making the CSE-correction effect negligible.

The reduction of CSE is possible via optimization of the SIP–nanoSIMS experimental conditions (*D*_*gs*_, *D*_*f*_, $N_{12_{C}}$ and $N_{13_{C}}$) in order to achieve minimal *CSV*_*K*_*A*__ and *CSH*_*K*_*A*__. Increase of raster density and current of primary ions (PI) improve the counting statistics. However, the increase of PI current causes also the reduction in focus quality and may render the derived isotope-ratio values unreliable when the counting of major isotopes reaches saturation. Thus, a compromise in SIP–nanoSIMS conditions has to be found to deliver heterogeneity values with acceptable errors; that is, HC_*corr*_ ± Δ HC_*corr*_ (SI, Supplementary Figures S1, S2).

The HC calculation developed in the present study for nanoSIMS-derived data, including CSE correction, were incorporated in the up-to-date version of the supplementary Excel template (Supplementary Table S1). The developed HC expression can be applied as a measure of heterogeneity not only of cellular anabolic activity, but also of any parameter measured at the single-cell level within a population: for example, length, volume, fluorescence yield, and gene expression (Nikolic et al., 2013; Gangwe Nana et al., 2018; Simşek and Kim, 2018; Heyse et al., 2019). The supplementary Excel template was therefore extended for HC calculation on the data acquired with other single-cell-resolved techniques.

### Dispersion of Metabolic Activity in Monoclonal Subpopulations

Different studies dealing with phenotypic heterogeneity have shown that a population splits in subpopulations with different functions and/or activities (Simpson et al., 2009; Arnoldini et al., 2014; Kotte et al., 2014; Lieder et al., 2014; Li et al., 2018; Simşek and Kim, 2018). The same outcome was found in our results, in particular for *P. putida* strain. The histograms of *K*_*A*_ distribution of both strains investigated here (Figures 3B,C) revealed several peaks (subpopulations) with different centroids of anabolic activity. For example, for *P. putida* cells after 60 min of incubation (Figure 3C), several subpopulations could be clearly resolved in the histogram (with ${\tilde{K}}_{A}$ at around 0.03, 0.27, 0.48, 0.76, and 0.95). Even without considering the outer two subpopulations, the cell number in the first three subpopulations is comparable, rendering the overall *DW* of the entire distribution not representative for the *K*_*A*_ dispersion of the single subpopulations. Also, the consideration of a single centroid value $\left({\tilde{K}}_{A}\right)$ renders the HC calculation inappropriate when the cell activity is distributed over several subpopulations. Thus, a reliable approach for quantitation of heterogeneity upon multimodal anabolic activity is necessary, ensuring the elucidation of (i) the activity dispersion in monoclonal subpopulations and (ii) its propagation into the entire microbial population.

Empirical approximation can be applied to study the relation between cellular properties, surrounding environments and experimentally observed development of heterogeneity in single-cell functions. Empirical analysis has been employed in allometry, for example, to describe the relation between animal body mass and its metabolic rate with the power function according to Kleiber's law (Kleiber, 1947). Another example of empirical approximation is the relation between cell dry mass and its volume described with a power function (Loferer-Krössbacher et al., 1998).

Furthermore, the distribution of single-characteristic values ranked in their descending order has been also described with the power function. George K. Zipf had shown (Equation 23) (Zipf, 1935) that the frequency (*f*) of word appearance in a text of natural language is inversely proportional to the rank (*r*) of a word in the word frequency table sorted in frequency descending order (Powers, 1998),

(23) $$\begin{array}{l} f(r;s,N)=\frac{\frac{1}{r^{s}}}{\sum_{n=1}^{N}\left(\frac{1}{n^{s}}\right)} \end{array}$$

where *N* is the number of words in a text.

The power function *f*(*r*; *s, N*) Equation (23) represents the Zipf's law and thanks to the variable power index (*s*) is applicable in different fields whenever the power law is obeyed. George U. Yule had applied the analysis of rank–frequency distribution to study new genera development from monotypic genus during evolution (Yule, 1925). Power law approximation has been employed also in ecological applications to quantify the relative abundance of species in different ecosystems (Mouillot and Lepretre, 2000). The statistical properties of selfish spreading DNA repeats were also studied with a power-low approach, explaining how the high abundance of long DNA elements does not depend on the coded functions but rather on the ability of selfishly spreading in the host genome (Sheinman et al., 2016). The rank–frequency distribution of guanine/cytosine nucleotides in mitochondrial DNA has been approximated with Zipf's law to distinguish families and genera for taxonomic classification (Rovenchak, 2018).

In the present study, we applied the power law approach (i) to measure the ability of single cells to differentiate from each other in their anabolic activity and (ii) to account for the subpopulations inside a monoclonal population. The derivation of the *s* index in single-cell rank–activity distribution allowed for quantitation of anabolic heterogeneity as explained below.

#### Differentiation Tendency in Cellular Anabolic Activity

The development of metabolic heterogeneity was resolved with the rank–activity distribution of single cells plotted in double-logarithmic scale. The analyzed cells were ranked according to their anabolic activity (*K*_*A*_) sorted in descending order; the cells with a low *r* show higher anabolism as compared to those at high *r* ranges.

**Figure 4:**
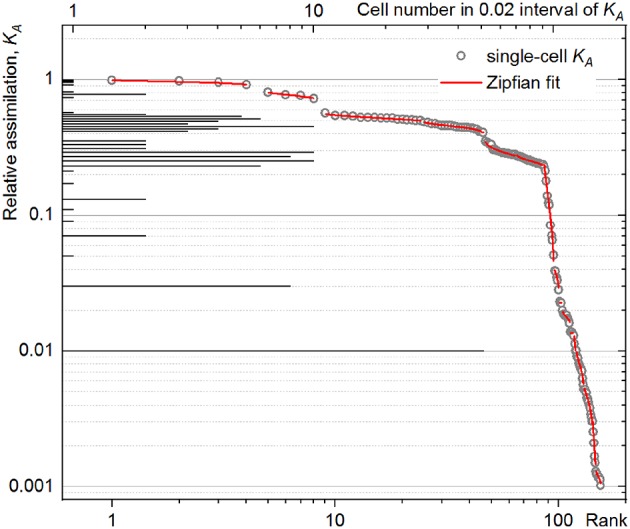
Rank–activity distribution (bottom *X*-axis) of *Pseudomonas putida* single cells after 60 min of incubation with an isotope-labeled growth substrate. The relative assimilation of each single cell is represented in the rank–activity plot with hollow circles. For comparison, the corresponding histogram from Figure 3 was overlaid (top *X*-axis).

The rank–activity plot of *P. putida* cell at 60-min time point (Figure 4, open circles) shows the distribution of single cells along multiple steps. Each step shows a specific slope describing the tendency of the cells to differentiate in their anabolic activity. This differentiation tendency of single cells causes the population heterogeneity and can be measured for each subpopulation by the steepness of the corresponding slope in the rank–activity distribution. In the present study, common trend in the activity differentiation, that is, fitting a single slope, was considered as criterion for assignment of single cells to a certain subpopulation. The *K*_*A*_ distribution slope revealed in the double-logarithmic coordinates plot (log(*K*_*A*_); log(*r*)) can be described with *s* exponent of a power function as:

(24) $$\begin{array}{l} \;K_{A}\propto C\times r^{-s};\;\;C\equiv \text{constant}\\ \log(K_{A})=\log(C)-s\times \log(r) \end{array}$$

predicting the anabolic activity (*K*_*A*_) of single cells with their rank *r* in the cells series sorted in *K*_*A*_ descending order.

The *K*_*A*_ stays constant (*K*_*A*_ = *C*) with the power index *s* = 0 (Equation 24) describing the case in which cells grow without differentiation in their anabolism and their heterogeneity is approaching zero. Higher power index values *s* indicate a steeper slope of rank–activity distribution, implying an increase in the differentiation of anabolic activity and a heterogeneity gain. Thus, the power index *s* was considered as a measure of anabolic heterogeneity and is referred hereafter as DTI (*s*).

#### Quantitation of Differentiation Tendency in Anabolic Activity

Rank distributions of real experimental data, following a power law, show usually a single slope in the range of intermediate *r* values, but its profile in the range of low and high *r* values shows a considerable deviation from a single slope. Numerous modifications of the Zipfian function (Yule, 1944; Mandelbrot, 1953; Simon, 1955; Lavalette, 1996) have been suggested for the improvement of experimental data fit at either low or high *r* values. Poor fit accuracy and lack of meaningful parameters' interpretation limit the application of power law functions for approximation of experimental data. In the present study, the expression of the Zipfian function Equation (25), previously suggested by Voloshynovska for the analysis of word frequencies in texts (Voloshynovska, 2011), was adopted for the approximation of the rank–activity distribution of our experimental data (Figure 4):

(25) $$\begin{array}{l} K_{A}\left(r;q,s,N\right)=C\times {\left(\frac{N\times \left[r+q\right]}{\left(N-\left[r+q\right]+1\right)}\right)}^{-s};\;C\equiv \text{constant}. \end{array}$$

The *r* + *q* term, suggested by Mandelbrot, provides the possibility to improve the Zipfian fit in the low-rank range (small *r* and high *K*_*A*_ values). Lavalette's term $\left(\frac{N}{N-r+1}\right)$ facilitates a more accurate fit of rank–activity distribution in the high *r* range (high *r* approaching *N* and low *K*_*A*_ values) and takes explicitly the size (*N*) of a population (or a subpopulation) into account.

In the case of cell rank–activity distribution, the *s* in the power exponent (Equations 24 and 25) stays invariant to linear normalization and to population size (Voloshynovska, 2011), allowing therefore for the following:

- comparison of heterogeneity in subpopulations with different ${\tilde{K}}_{A}$ centroids;
- quantitation of differentiation tendency in anabolic activity of subpopulations with low cell abundance;
- resolution of subpopulations possessing close median values; and
- elucidation of differentiation in anabolic activity even after a short incubation with stable-isotope-labeled growth substrates.

Importantly, the CSE propagation does not influence the slope of rank–activity distribution as it does on the DW considered in the HC expression (Equation 6). Therefore, DTI is less sensitive to the *CSV*_*K*_*A*__ variation (Equation 18) as long as *D*_*f*_ is kept well below *D*_*gs*_ and a sufficient number of isotope ion counts is accumulated during the data acquisition with nanoSIMS; otherwise, high *CSV*_*K*_*A*__ value smears the rank–activity distribution and may enhance the uncertainty in the slope determination, lowering the accuracy of the experimental data fit (Figure 4) considered below.

For a unimodal anabolic activity, the rank–activity distribution was expected to show a single slope, namely, without a subpopulation outcome. To underpin this assumption, simulated normal distributions (Supplementary Figures S3A, S4) were approximated with the Zipfian function (Equation 25). Normal distributions were simulated, keeping the mean value fixed and changing the σ values; then DTI and CV values were calculated for each of the distribution, revealing the same trend in σ (Supplementary Figure S3B). This behavior of DTI provided proof of its suitability as a heterogeneity index under unimodal activity. Moreover, the Zipfian approximation delivers the slope values *s* = 0.1624 ± 0.0006 for 1,000 cells, *s* = 0.1631 ± 0.0011 for 200 cells, and *s* = 0.1619 ± 0.0082 for 50 cells, proving the invariance of *s* (DTI) to the population size, as shown in Supplementary Figure S5.

For the instance in which the population is split in multiple subpopulations (multimodal distribution, Figure 4), the rank–activity distribution was implemented here with the *K*_*A*_(*r*; *q, s, N*) expression (Equation 25) represented as a combination of subpopulation activities *K*_*A*_*i*__ (*i*∈[ 1, *m*]):

(26) $$\begin{array}{l} K_{A}(r;q,s,d,n)=\overset{m}{\underset{\begin{array}{l} \;i\\ d_{i}\leq r<d_{i+1} \end{array}}{\sum^{\;}}}(d_{i}\leq r<d_{i+1})\times C_{i}\times \\ \;\begin{array}{l} \times {\left(\frac{n_{i}\times [|r-d_{i}+q_{i}|+1]}{\left(n_{i}-[r-d_{i}+q_{i}+1]+1\right)}\right)}^{-s_{i}} \end{array} \end{array}$$

*m*: number of subpopulations;

*d*_*i*_: rank of cells with the highest activity in a subpopulation (*i* ∈ [ 1, *m*]);

*C*_*i*_: scaling constant of the activity in a subpopulation (*i* ∈ [ 1, *m*]);

*n*_*i*_: number of cells in a subpopulation (*i* ∈ [ 1, *m*]);

*q*_*i*_: Mandelbrot's parameter for a subpopulation (*i* ∈ [ 1, *m*]);

*s*_*i*_: slope of rank–activity distribution in a subpopulation (*i* ∈ [ 1, *m*]);

*r*: rank (*r* ∈ [ 1, *N*]);

*N*: total number of cells in the whole population $\left(N=\overset{m}{\underset{i=\;1}{\sum }}n_{i}\right)$.

The approximation of an experimental rank–activity distribution with *K*_*A*_(*r*; *q, s, d, n*) in this multicomponent function (Equation 26) provides a set of slope values *s*_*i*_, each of them characterizing the DTI in the anabolic activity of a subpopulation. In order to obtain a unique value, able to describe how the differentiation in anabolic activity of single subpopulations is propagated into the entire monoclonal population, the CDTI (*S*) was introduced. The CDTI was calculated as the distance in the *m*-dimensional Euclidean space considering *s*_*i*_ values of *m* subpopulations as differentiation propagation vectors using the following expression:

$$\begin{array}{l} S=\sqrt{\overset{m}{\underset{i=1}{\sum }}{\left(\gamma_{i}\times s_{i}\right)}^{2}}\;\left(i\in \left[1,m\right]\;\right). \end{array}$$

The coefficients γ_*i*_ were introduced to weight the contribution of each subpopulation into the cumulative differentiation. The weighting term γ_*i*_ is expressed as a logarithm of a cell number (*n*_*i*_) in a subpopulation relative to the logarithm of the total number of cells in the whole population (*N*).

$$\begin{array}{l} \gamma_{i}=\frac{\log\left(n_{i}\right)}{\log\left(N\right)}\;\left(i\in \left[1,m\right]\right) \end{array}$$

Taking the weighting term into account, the CDTI was expressed in the following way:

(27) $$\begin{array}{l} S=\sqrt{\overset{m}{\underset{i=1}{\sum }}{\left(\frac{\log\left(n_{i}\right)}{\log\left(N\right)}\times s_{i}\right)}^{2}}\;\left(i\in \left[1,m\right]\right). \end{array}$$

The propagation of the fit errors Δ*s*_*i*_ into the calculated CDTI (Equation 27) results in Δ*S* expressed as

(28) $$\begin{array}{l} \Delta S=\sqrt{{\sum^{\;}}_{i=1}^{m}{\left(\frac{\partial S}{\partial s_{i}}\times \Delta s_{i}\right)}^{2}}=\sqrt{\frac{\sum_{i=1}^{m}{\left[\gamma_{i}{\;}^{2}\times s_{i}\times \Delta s_{i}\right]}^{2}}{\sum_{i=1}^{m}{\left(\gamma_{i}\times s_{i}\right)}^{2}}}. \end{array}$$

The CDTI calculations (*S* and Δ*S*) developed in the present study were implemented in the supplementary Excel template (Supplementary Table S2).

#### Power Law Fit of Rank–Activity Distribution Derived From NanoSIMS Data

**Figure 5:**
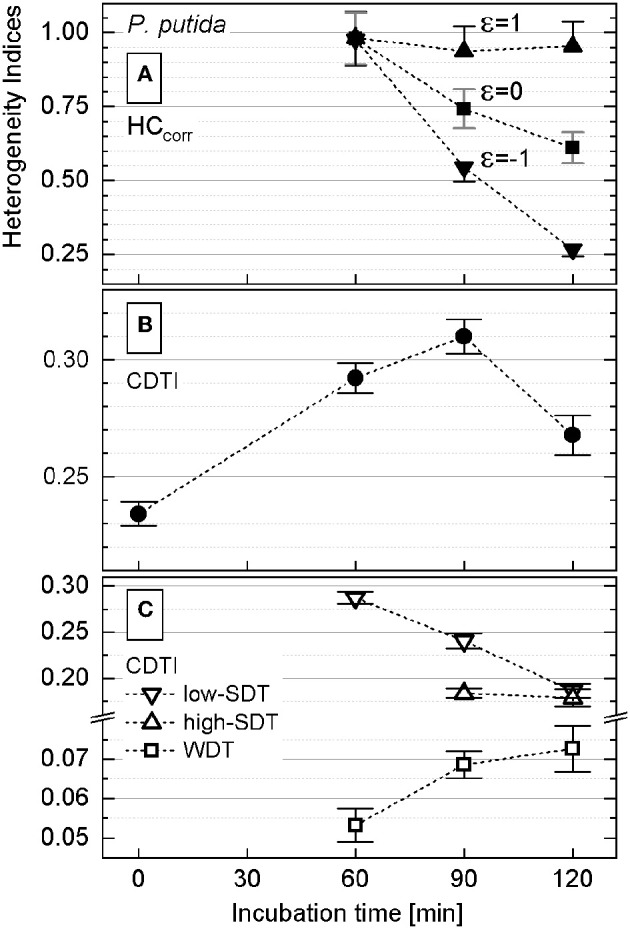
Heterogeneity indices derived for *Pseudomonas putida* after different incubation times. **(A)** Comparison of the HC_corr_ (Equation 21) calculated with different ε values; error bars represent the ΔHC_corr_ (Equation 21′). **(B)** Cumulated differentiation tendency index (CDTI) trend of entire populations (represented as *S* ± Δ*S*; Equations 27 and 28); error bars show the ±Δ*S* intervals. **(C)** CDTI calculated (Equations 27 and 28) for cell subpopulations revealing weak differentiation tendency (WDT) and strong differentiation tendency at low/high *K*_*A*_ (low/high SDT).

The rank–activity distributions of *P. putida* cells after different incubation times were approximated with the multicomponent Zipfian function (Equation 26; Supplementary Figures S6A, S7). First, DTI (*s*_*i*_ ± Δ*s*_*i*_) values were derived as fitting parameters for all the revealed subpopulations; then the CDTI (*S* ± Δ*S*) values (Equations 27 and 28; Supplementary Table S2) were calculated and compared with the corresponding HC_corr_ ones (Figure 5).

Even when calculated with ε ∈ [−1;1] (Equation 21), the HC_corr_ (Figure 5A) did reveal neither a clear correlation nor a common trend with the corresponding CDTI (Figure 5B) for *P. putida*. To clarify this discrepancy, the rank–activity plot was overlaid with the corresponding histogram (Figure 4). The representation of *K*_*A*_ distribution with a rank–activity plot allows for distinction between cells with close *K*_*A*_ values, providing a single-cell resolution, which is not achievable with a histogram plot. The profiles of rank–activity distribution (Figure 4; Supplementary Figures S7, S9) show the cells dispersed into two main groups. In general, the cells in the lower-rank range (high *K*_*A*_ values) are fitting moderate slopes, whereas those in the high-rank range show considerably steeper slopes. Thus, the population is clearly split into two cell–activity trends: (i) weak differentiation tendency (WDT) and (ii) strong differentiation tendency (SDT). The SDT cells (steeper slopes) can also be observed in the lower-rank range (high *K*_*A*_ values) as revealed by *P. putida* populations at time points of 90 and 120 min (Supplementary Figure S7). The SDT cells showing high anabolic activity (high *K*_*A*_ values) are metabolically different from those showing low anabolic activity (low *K*_*A*_ values). Therefore, high-SDT cells (high *K*_*A*_ values) were differentiated from low-SDT cells (low *K*_*A*_ values), and the CDTI values were calculated separately for the corresponding SDT subpopulations of *P. putida* at 90- and 120-min time points (Figure 5C). At a time point of 60 min, *P. putida* populations did not show high-SDT cells; consequently, the CDTI values were calculated for WDT and low-SDT cells. Considering the CDTI calculated separately (Figure 5C), the SDT subpopulations reproduced the descending trend of the HC_corr_ derived with ε ≤ 0 (Figure 5A), providing the major contribution to the heterogeneity of the entire population (CDTI, Figure 5B).

**Figure 6:**
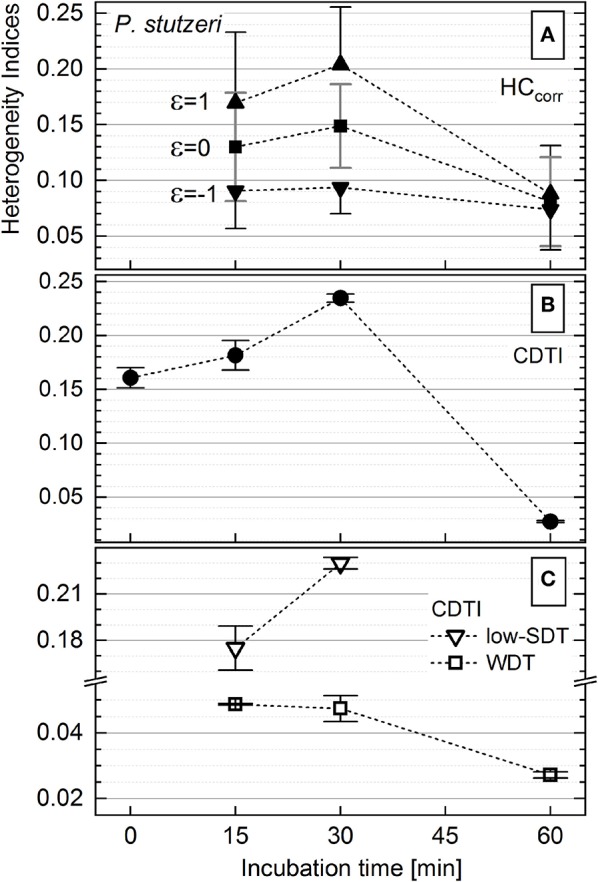
Heterogeneity indices derived for *Pseudomonas stutzeri* after different incubation times. **(A)** Comparison of the HC_corr_ (Equation 21) calculated with different ε values; error bars represent the ΔHC_corr_ (Equation 21′). **(B)** Cumulated differentiation tendency index (CDTI) trend of entire populations (represented as *S* ± Δ*S*; Equations 27 and 28); error bars show the ±Δ*S* intervals. **(C)** CDTI calculated (Equations 27 and 28) for cell subpopulations revealing weak differentiation tendency (WDT) and strong differentiation tendency at low *K*_*A*_ (low SDT).

The activity of *P. stutzeri* single cells (Figure 3B), contributing to the main peak in the histogram, was considered to be unimodal. Those cells were ascribed to the WDT subpopulation, and their rank–activity distribution was initially approximated with the single-component Zipfian function (Equation 25), delivering DTI as *s* ± Δ*s* values. However, this approximation provided considerably high relative errors (Δ*s*/*s* × 100 [%]; Supplementary Figure S8). Consequently, the rank–activity distributions of entire *P. stutzeri* populations were approximated (Supplementary Figures S6B, S9) with the multicomponent Zipfian function (Equation 26), and the CDTI values were derived as *S* ± Δ*S* (Supplementary Table S2) according to Equations 27 and 28 (Figure 6B). The low-SDT and WDT subpopulations were recognized in the rank–activity distribution, and the corresponding CDTI values were calculated separately (Figure 6C). The increase in CDTI from 15- to 30-min time points is driven by low-SDT subpopulations (Figure 6C, triangles), which correspond to the single cells showing low *K*_*A*_ values in the histogram (Figure 3A). Those cells are included into the HC_corr_ calculation when ε ≥ 0 (Figure 6A), resulting in the HC trend following the one revealed by CDTI (Figure 6B) for the entire *P. stutzeri* population. Hence, the SDT subpopulations were shown to contribute mostly to the overall heterogeneity in anabolic activity of *P. putida* and *P. stutzeri*. The CDTI trends over time of both *Pseudomonas* strains are compared in Supplementary Figure S10.

As mentioned in Correction of the HC for the CSE, the calculation of HC on SIP–nanoSIMS data is unreliable for T0 when small changes in cellular heavy-isotope enrichment (small *R*_*f*_ – *R*_*i*_ difference in Equation (1); *K*_*A*_ → 0) are considered and cellular heavy-isotope content is close to the natural abundance. Instead, rank–activity plot in double-logarithmic coordinates resolves also the low-abundance subpopulations (i.e., a slope can be defined with three cells already), allowing for calculation of CDTI and therefore the quantitation of heterogeneity prior to labeling or at a very short incubation time. The results of multicomponent Zipfian fit of rank–activity for both strains at T0 are presented in Supplementary Figure S6. The corresponding *S* values (Supplementary Table S2) were introduced into the CDTI plots for both strains (Figures 5B, 6B; Supplementary Figure S10) to elucidate the development of heterogeneity, expressed as CDTI, starting from T0. Thus, at T0, both *Pseudomonas* populations show already anabolic heterogeneity, revealing multimodality; afterwards, each species develops distinct tendency in heterogeneity according to a strain-constitutive strategy.

### Method Applicability

To demonstrate the broad applicability of the suggested heterogeneity indices, HC and DTI/CDTI were derived for two different datasets: (i) the distribution of DAPI-stained single cells of *P. putida* acquired with flow cytometry and (ii) the distribution of *Escherichia coli* single-cell length upon different growth conditions reported by Nikolic et al. (2017).

#### Heterogeneity Quantitation on Flow Cytometry Data

**Figure 7:**
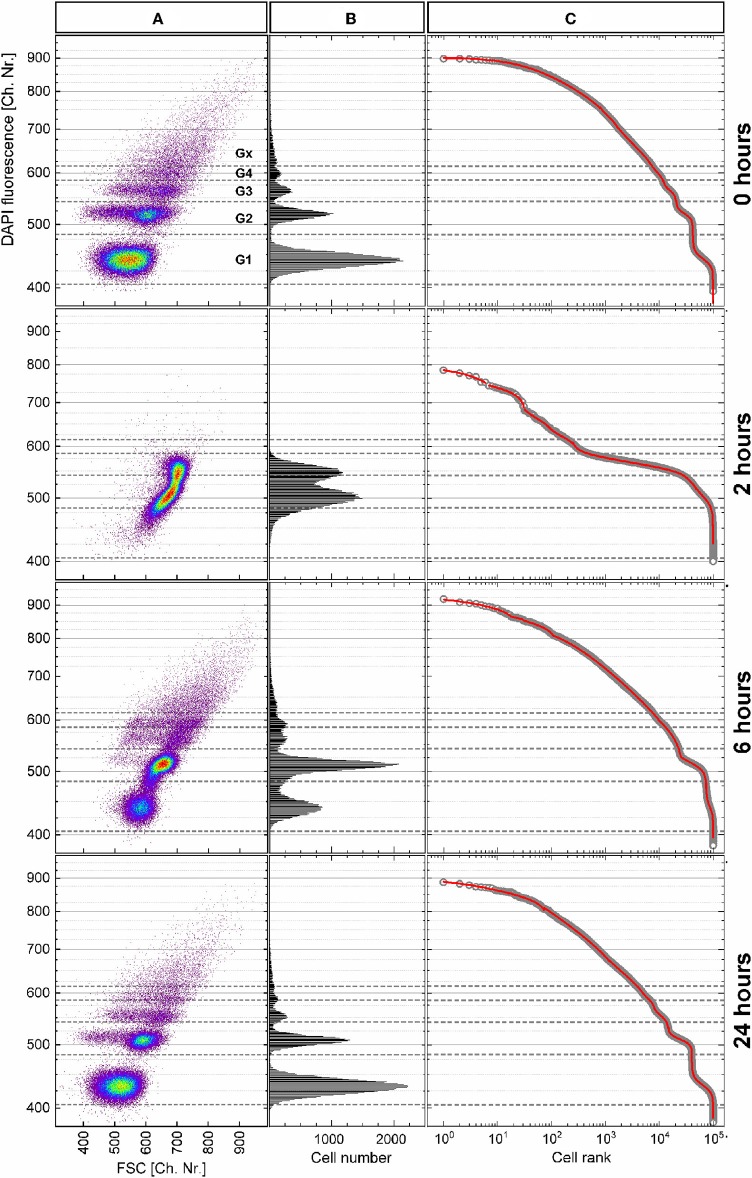
Heterogeneity dynamics of *Pseudomonas putida* population during growth over 26 h. **(A)** DAPI fluorescence intensity (related to DNA content) vs. forward scatter (FSC) intensity (related to cell size) dot plots of the 0-, 2-, 6-, and 24-h samples after data transformation (details about the data transformation and dot plots of the remaining samples in Supplementary Figures S11, S12). **(B)** Histograms of DAPI fluorescence intensity distribution used to define the boundaries between the subpopulations G1–Gx, which correspond to the chromosome number in the cells. **(C)** Cells ranked according to their fluorescence intensity (open circles) together with the multicomponent Zipfian fit (solid red line).

The *P. putida* cells were analyzed with flow cytometry at eight time points upon cultivation in batch for 26 h. The cellular DNA content was measured as DAPI fluorescence intensity and visualized in dot plots (Figure 7A; Supplementary Figure S11) together with the cell-size-related FSC intensity. The cells were distributed within a subpopulations' pattern that changed dynamically during cultivation. The boundaries between the five subpopulations G1, G2, G3, G4, and Gx were defined with this pattern according to the local minima in the DAPI fluorescence intensity histogram (Figure 7B) at inoculation (0 h). The distribution of cells along this histogram represents the different chromosome numbers per cell that can occur during different states of cell growth. During the cell cycle, cells increase in size (frequently, but not always) while duplicating their DNA, and consequently, their FSC intensity increases (Müller, 2007).

**Figure 8:**
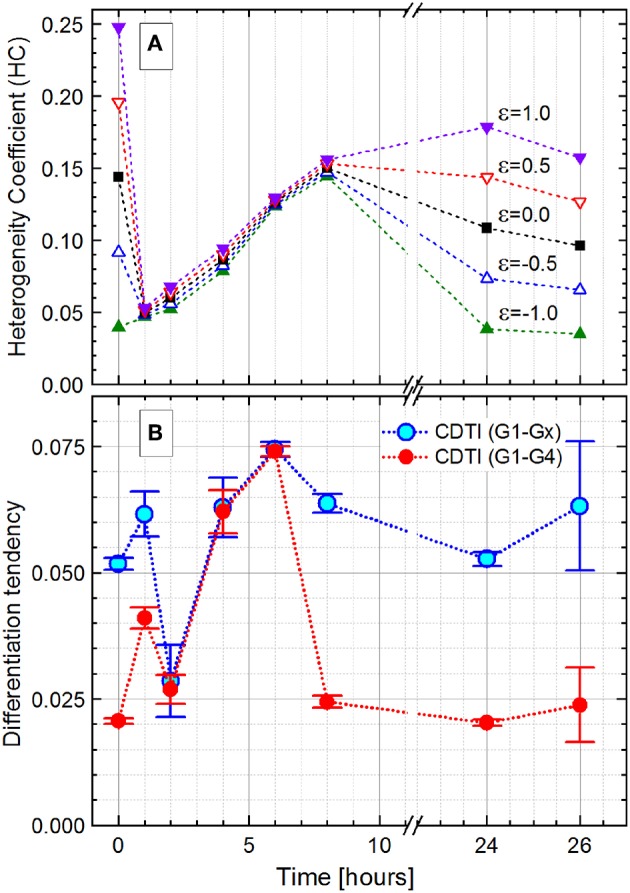
Development of the *Pseudomonas putida* culture heterogeneity over the 26-h growth expressed as the heterogeneity coefficient (HC) with different ε values (Equation 16; **A**) and cumulated differentiation tendency index (CDTI, **B**). In **(B)**, CDTI_G1−Gx_ for the entire population and CDTI_G1−G4_ for cells in G1 to G4 subpopulations are represented as *S* ± Δ*S* (Equations 27 and 28); because of small Δ*S* values (Δ*S*/*S* ≤ 0.001), the magnified ±Δ*S* × 500 intervals are shown with the error bars.

The observed changes in cellular DNA contents result in heterogeneous distributions that can be described by the HC index and the CDTI. For this purpose, the flow cytometric raw data in FCS 3.0 format (Seamer et al., 1997) were treated as described in Supplementary Figure S11. In short, the values in logarithmic scale were translated into equally distributed 1,024 channels. In a next step, only the range of channels that represented cells was plotted. Finally, the cells were ranked according to their DAPI fluorescence intensity, and the multicomponent Zipfian function (Equation 26) was fitted to the resulting rank–distribution curves (Figure 7). The changes in the Zipfian slope (Figure 7C) matched the established subpopulation segregation, deduced from the dot plots (Figure 7A) and shown by the corresponding histograms (Figure 7B). The heterogeneity in cellular DNA content was expressed with the HC index (despite the multimodality) and the CDTI calculated (Supplementary Table S2) with the results of multicomponent Zipfian approximation. The development over the 26-h growth curve of both indices is represented in Figure 8.

For the initial and final growth time points, the HC was highly dependent on the ε value, which can be used to adjust the index sensitivity to the distribution skew. The HC decreased from the time of inoculation, when the population is constituted by cells with varying chromosome numbers, to the 1-h time point with most cells containing only one chromosome. During cell growth, the HC showed the heterogeneity boost due to an increasing number of cells harboring two, four, and more chromosomes.

Interestingly, the HC values do not change much with the variation of ε values during the exponential phase. The ε value weights the asymmetry (skew) contribution in the HC. The reduced effect of ε can be considered as an evidence for a negligible skewness. In the case of multimodality, a small skewness value may be achieved with the centroid (median, Q_2_), located equidistant between two quantiles (Q_1_ and Q_3_), even though a single *Q*_2_ value is not representative in the case of a multimodal distribution. Finally, at the 24-h time point, with the population in the stationary phase, the HC values differentiate according to the chosen ε values again. In the present study, the error in graded fluorescence intensity was estimated to be 2 of 1,024 channels (<0.5%) and therefore neglected upon the HC calculations.

The HC describes the general dynamic of heterogeneity during the population growth, but its explanatory power is restricted in case of subpopulation segregation displayed by the *P. putida* culture. In this case, the CDTI is more suitable for the heterogeneity quantitation since it accounts for multimodality. The CDTI_G1−Gx_ was initially calculated for the entire population with all slope values derived from the Zipfian fit (Figure 8B, rectangles). In order to avoid overweighting the influence of low-probability events in Gx that likely comprise cell aggregates (0 h, 6.60%; 1 h, 0.45%; 2 h, 0.18 %; 4 h, 4.44%; 6 h, 6.80%; 8 h, 9.49%; 24 h, 6.00%; and 26 h, 3.28%), the CDTI_G1−G4_ (Figure 8B, circles) was additionally calculated for the cells in the clearly segregated subpopulations G1–G4. The CDTI_G1−G4_ value increased from 0 to 1 h when cells started to duplicate their chromosomes after a short lag phase. At 2 h, most cells have doubled their DNA content and subsequently start the duplication resulting in (i) the majority of cells having a single chromosome and (ii) heterogeneity decrease. In the exponential growth phase, the population showed increasing CDTI_G1−G4_ at 4 and 6 h with higher chromosome numbers and uncoupled DNA synthesis. The confinement of cell distribution in the G1–G4 subpopulations resulted in the reduction of CDTI_G1−G4_ at 8 h when the culture gets into the stationary phase. In the advanced stationary phase at 24 h, the cell distribution pattern is very similar to the one upon inoculation (0-h time point). This pattern similarity is supported by the close CDTI_G1−G4_ values derived for 0- and 24-h time points. The restarting cell activity after the acetate feeding at 24 h was captured by the slight increase of CDTI_G1−G4_ at 26 h. Thus, the CDTI_G1−G4_ (Figure 8B, circles) describes the dynamics of the population heterogeneity, whereas the HC (Figure 8A) represents its general trend missing the resolution of subpopulations.

#### Heterogeneity Quantitation on Cell Length Distribution

**Figure 9:**
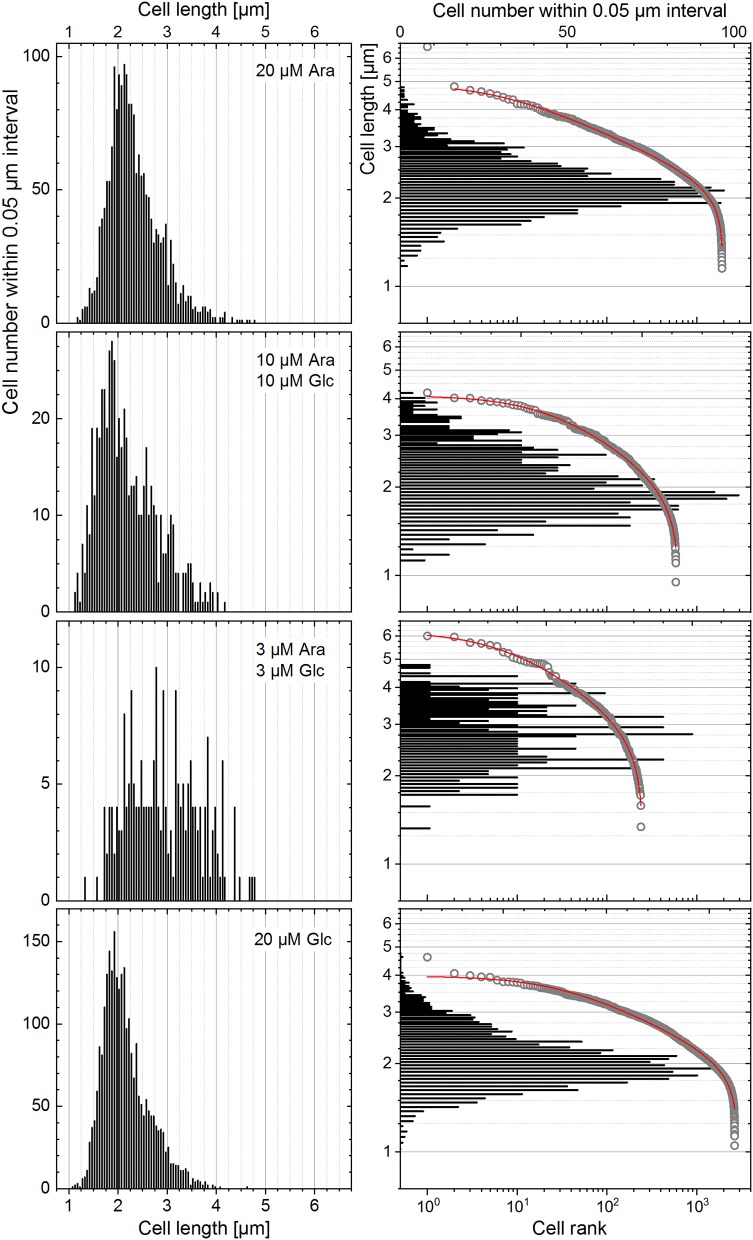
The distribution of single cells in their length represented for the different growth conditions reported in Nikolic et al. (2017) with histograms and rank–length plots.

**Figure 10:**
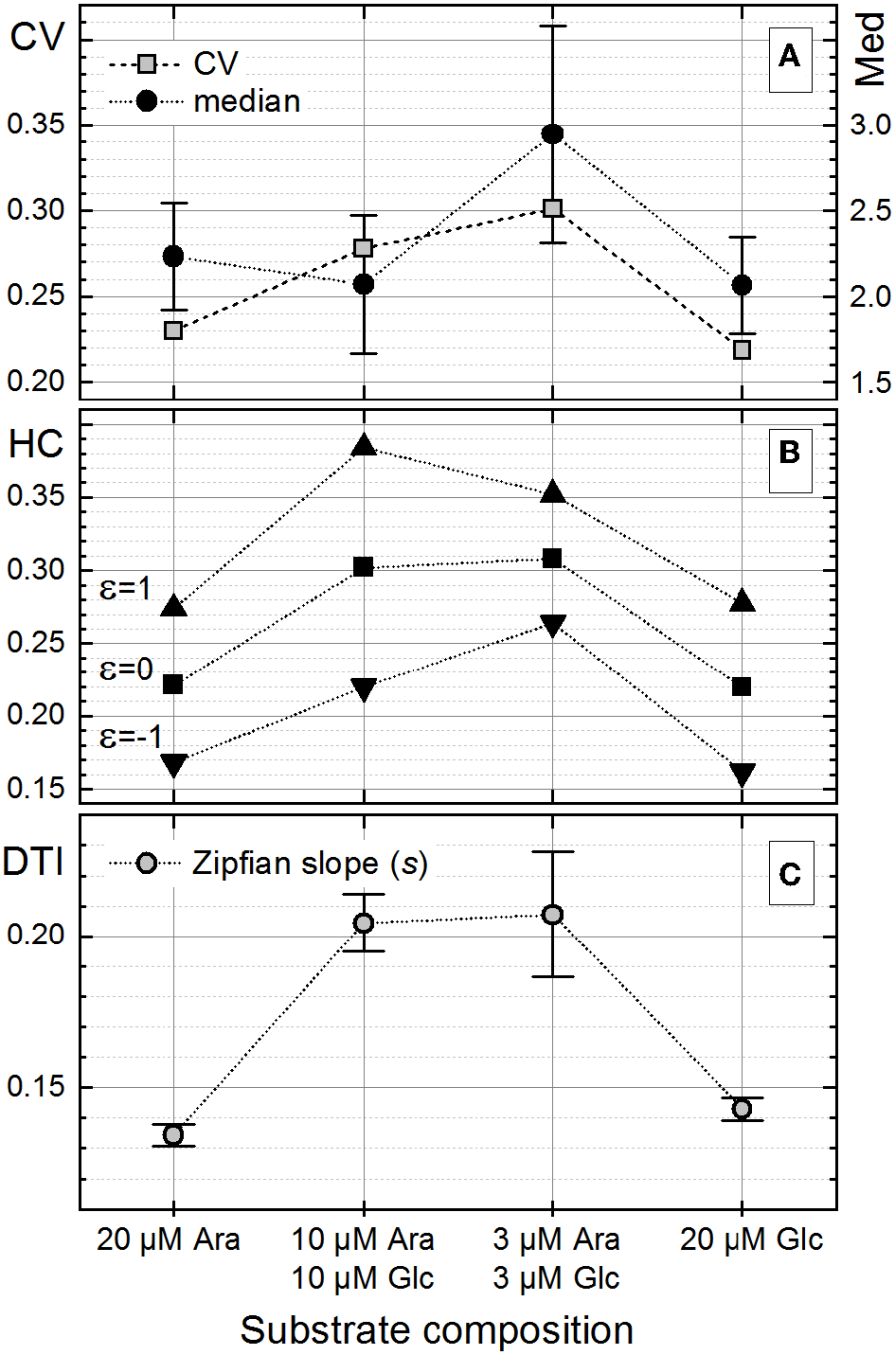
Changes in the single-cell length heterogeneity of *Escherichia coli* upon the different growth conditions reported in Nikolic et al. (2017). **(A)** The coefficient of variation (CV) together with the median of cell length [error bars represent the ±median absolute deviation (MAD) interval, Equation (7)]. **(B)** The heterogeneity coefficient (HC) (Equation 16) calculated with different ε values. **(C)** The Zipfian slope [differentiation tendency index (DTI)] of the rank–length distributions as *s* ± Δ*s* (Equation 25); because of small Δ*s* errors (Δ*s*/*s* ≤ 0.001) obtained with the single-component Zipfian approximation, the magnified ±Δ*s* × 100 intervals are shown with the error bars.

The heterogeneity quantitation was also applied to cell length distributions from a published dataset (Nikolic et al., 2017). In their work, *E. coli* cells, grown in the presence of two different carbon sources, did not specialize in a bimodal fashion but rather followed unimodality, consuming both substrates simultaneously at different rates. The highest degree of heterogeneity of the monoclonal population (measured with CV) was found during co-feeding with glucose (Glc) and arabinose (Ara) under carbon limitation as compared with the growth with one single carbon source under nitrogen limitation (Nikolic et al., 2017). This behavior was a consequence of different gene expression and transcription levels as well as different rates of single-cell growth in chemostat. Nutrient-dependent changes in the cultivation environment influence the growth rate, which in turn influences the cell size (Chien et al., 2012; Westfall and Levin, 2018). Bacterial cells have to find a compromise between the maintenance of a certain DNA amount and their cytoplasmic size in order to keep the DNA-to-cell mass ratio constant upon different growth rates. However, when external conditions start to be unfavorable, while cells continue to duplicate their DNA via multiple duplication forks, the mechanism of division via the FtsZ ring and other intracellular molecules is inhibited, thus resulting in a larger size of cells (Chien et al., 2012). The data on cell length provided by Nikolic et al. (2017), confirmed these experimental observations. This dataset was analyzed (Figure 9) to elucidate the heterogeneity in cell size upon different conditions reported in their study (Nikolic et al., 2017). The derived CV, HC index, and DTI are shown in Figure 10. The highest median value of cell length (Figure 10A, circles) was found with the “3 μM Ara + 3 μM Glc” substrate composition, which represents the strongest carbon limitation. The HC value calculated with −1 ≤ ε ≤ 0 (Figure 10B) followed the CV trend (Figure 10A, rectangles), as expected for unimodal distribution. The approximation of rank–length distributions with the single-component Zipfian function delivered the slope values (*s*, DTI; Figure 10C), bringing two interesting outcomes: (i) the heterogeneity of population in cell size upon “3 μM Ara + 3 μM Glc” is not much higher than that upon “10 μM Ara + 10 μM Glc” as revealed with HC (ε = 0; Figure 10B) although this difference was overestimated with the CV (Figure 10A) and (ii) the length of single cells showed a unimodal distribution confirmed by small Δ*s* (and high goodness of fit), which is in agreement with the unimodality reported in Nikolic et al. (2017).

### Guidelines on the Heterogeneity Quantitation

The summary of the approaches (HC and DTI/CDTI) developed in this study for heterogeneity quantitation is provided in Table 2. An accurate quantitation of cellular anabolic activity requires a reliable data acquisition with single-cell resolution. The aspects described for the SIP–nanoSIMS experiment (step 1 in Table 2) are, in general, relevant for experiments implying data acquisition in counting mode.

**Table 2:** Summary of heterogeneity quantitation approaches.

**Heterogeneity quantitation steps**	**Tools applied**
1.Acquisition of data with a single-cell resolution technique
• Stable isotope probing (SIP) with nanoscale secondary ion mass spectrometry (nanoSIMS) (SIP–nanoSIMS) experiment (*as a specific case*) 1.1. SIP: the dynamic range for cellular isotope enrichment (*D_*f*_*) is defined by isotope label content in the growth substrate (*D_*gs*_*); relative assimilation with acceptable error (*K_*A*_* ± Δ*K_*A*_*) is achieved with *D_*f*_* ≤ 0.6 × *D_*gs*_* (§3.1.3) 1.2. nanoSIMS: maximal precision of single-cell isotope content and pixel-average ion counts per cell can be achieved with the optimization of nanoSIMS conditions (SI §1, Supplementary Figures S1, S2). *Generally valid for data acquisition in counting mode*	• Look@NanoSIMS (LANS) software package for nanoSIMS data processing
2. Calculation of the heterogeneity coefficient (HC; §3.1)
2.1. Transfer of single-cell-resolved data into the template table S*pecific case*: Calculation of single-cell relative assimilation (*K_*A*_* ± Δ*K_*A*_*) with corresponding isotope ratio values (*R_*i*_, R_*f*_*) derived from nanoSIMS data (Equation 1)	• Excel template (Supplementary Table S1)
2.2. Calculation of HC (Equation 16; §3.1.2)	
2.3. Evaluation of counting statistics heterogeneity (CSH) for the applied conditions of, for example, SIP–nanoSIMS (Equations 17–19; §3.1.3)	
2.4. **IF** the CSH value approaches HC, **THEN** the HC_corr_ ± ΔHC_corr_ (Equations 21 and 21′; §3.1.3) corrected for counting statistics error has to be considered	
3. Quantitation of **Differentiation Tendency** [derivation of differentiation tendency index (**DTI**)**/cumulative DTI (CDTI)**; §3.2]
3.1.Plot of rank distribution with single-cell values in double-logarithmic scale	• OriginPro 2019
3.2. Approximation of rank–activity distribution with single-component Zipfian (Equation 25; §3.2.2)	
3.3. Evaluation of the rank–activity distribution slope (DTI, *s* ± Δ*s*) and the accuracy of single-component Zipfian fit	
3.4.**IF** a poor accuracy of the approximation with single-component Zipfian or large error (Δ*s*) of slope fit is achieved, **THEN** 3.4.1. Approximation of rank–activity distribution with multicomponent Zipfian (Equation 26; §3.2.2) 3.4.2. Calculation of the CDTI (*S* ± Δ*S*; Equations 27 and 28; §3.2.2)	• Excel template (Supplementary Table S2)

Each step in Table 2 contains a link to the corresponding section, equation, or figure in the main text or in **SI**. The calculation of HC including the CSE correction is implemented in the Excel template (Supplementary Table S1). The results of nanoSIMS data processing with the LANS software together with other input parameters (like *D*_*gs*_, *D*_*i*_, and *N*) have to be pasted into the appropriate green-marked fields, and the Excel template calculates the outputs including *K*_*A*_, HC, and HC_corr_ values. If the analyzed population of single cells follows unimodal anabolic activity, heterogeneity can be measured with HC or HC_corr_.

Unimodal rank–activity distribution can be also approximated with the single-component Zipfian function expressing the heterogeneity with the Zipfian slope as *s* ± Δ*s*. The approximation of multimodal rank–activity distribution with single-component Zipfian delivers poor fit accuracy, resulting in large Δ*s* values. In such a case, the rank–activity distribution has to be approximated with the multicomponent Zipfian function, and heterogeneity is measured with CDTI (*S* ± Δ*S*). The CDTI calculation was also implemented in the corresponding Excel template (Supplementary Table S2). In this study, the OriginPro 2019 software was used for Zipfian approximation of rank–activity distributions.

When heterogeneity has to be quantitated on a set of epifluorescence microscopy data, the error in the HC and DTI/CDTI indices may arise from the following factors: (i) non-linearity of fluorescence signal detection; (ii) contribution of fluorescence background; (iii) presence of contaminants and extracellular substances. These factors may modulate the fluorescence intensity affecting the resulting HC and DTI values. Both indices are influenced by background subtraction while the DTI remains invariant to linear normalization and cell number.

## Conclusions

In the present study, indices to measure metabolic heterogeneity were subjected to a comprehensive consideration. The expression of HC was developed for the quantitation of heterogeneity within a single-cell dataset. The adjustment of HC sensitivity to the distribution asymmetry was discussed and implemented in HC expression. When calculated with the same “sensitivity settings,” HC allows for heterogeneity comparison among different datasets. Importantly, the implemented HC expression returns the CV value if applied on datasets that reveal normal distribution. For the HC calculated from the nanoSIMS data, the correction for CSE was implemented. The influence of experimental conditions on CSE propagation into the calculated HC was discussed, and appropriate recommendations for a SIP–nanoSIMS experiment setup were made.

Anabolic heterogeneity of monoclonal populations can be also measured with the slope *s* of a rank–activity distribution, characterizing the tendency of cells to differentiate in their activity within several subpopulations. The DTI (*s*) is derived for each subpopulation as an exponent of the Zipfian power law approximation function. The CDTI (*S*) is derived from DTI values of a subpopulation set and measures the anabolic heterogeneity of the entire population. The power exponent *s* (DTI) is invariant to linear normalization and to the number of cells in a subpopulation/population. Both HC and DTI can be used as heterogeneity indices when a population shows unimodal anabolic activity. If the anabolic activity becomes multimodal, the DTI of each subpopulation (*s*_*i*_) has to be first derived with multicomponent Zipfian approximation, and thereafter, the calculation of CDTI (*S*) delivers a measure of anabolic heterogeneity of the entire monoclonal population. Besides SIP–nanoSIMS, the applicability of both developed indices was shown on flow cytometry and epifluorescence microscopy datasets. The HC and DTI/CDTI were proven to be robust indices for quantitative and comparative analyses of heterogeneity in single-cell-resolved studies. Thus, these indices are not restricted to particular techniques, have a broad range of applications, and measure phenotypic heterogeneity in all its aspects: metabolic, physiological, and morphological.

## Data Availability Statement

The flow cytometry dataset generated and analyzed in this study is available in the FlowRepository (ID: FR-FCM-Z2AS).

## Author Contributions

FC, IV, and HS conceived the developed methods and developed the quantitation of differentiation tendency (DTI/CDTI). FC, NM, FM, SM, HR, and HS designed the experiments. FC performed the growth experiments and isotope labeling and processed the nanoSIMS data. FC and HS performed the nanoSIMS analysis, developed the mathematical expression for the heterogeneity coefficient (HC), and wrote the manuscript with contribution from all authors. SM and JL performed the flow cytometry experiment. SM, FC, JL, and HS processed the flow cytometry data. MS, LW, SM, JL, and MT provided advices and critical inputs. All authors contributed to manuscript revision and approved the submitted version.

### Conflict of Interest

IV was employed by company le-tex publishing services GmbH. The remaining authors declare that the research was conducted in the absence of any commercial or financial relationships that could be construed as a potential conflict of interest.

## Abbreviations

SIP–nanoSIMS: stable isotope probing combined with nanoscale secondary ion mass spectroscopy
*K*_*A*_: fraction of an element incorporated by a single cell during the incubation with isotope-labeled growth substrates
CV: coefficient of variation (equal to the ratio of standard deviation to the mean)
CQD: coefficient of quartile deviation
COD: coefficient of dispersion
*Q*_*n*_: quantile with percentile *n*
*Sk*: skewness, indication of asymmetry in the distribution of a population
DW: distribution width on the histogram representing the distribution of a population
HC: heterogeneity coefficient developed in the present study
CSE: counting statistics error
HC_corr_: heterogeneity coefficient corrected for counting statistics error coming from nanoSIMS measurement
*CSV*_*R*_, *CSV*_*K*_*A*__: counting statistics variations of isotope ratios (*R*) and relative assimilation (*K*_*A*_)
*CSH*_*K*_*A*__: counting statistics heterogeneity of anabolic activity
DTI: differentiation tendency index defined as a slope (*s*) in a rank–activity distribution
CDTI: cumulative differentiation tendency index
SDT: strong differentiation tendency
WDT: weak differentiation tendency.
