# Supplementary material for: Quantitation and Comparison of Phenotypic Heterogeneity Among Single Cells of Monoclonal Microbial Populations
Source: Front Microbiol. 2019 Dec 20;10:2814. doi: 10.3389/fmicb.2019.02814 (PMC6933826; doi:10.3389/fmicb.2019.02814)
Supplement: Supplementary file 1 [file Data_Sheet_1.pdf]

1. Optimization of parameters for nanoSIMS measurement

In order to get a considerable precision in isotopic composition at single-cell level, the nanoSIMS measurement parameters were optimized prior the analysis of isotope-labelled samples.
P. stutzeri cells were prepared as explained in material and methods section for nanoSIMS analysis. Cells prior labelling were analyzed in order to avoid further effect of single cell variability in ^{13}C uptake. Therefore, single-cell precision of the derived isotope content was evaluated at the level of natural ^{13}C abundance.

Analysis of single-cell isotopic content was performed using a NanoSIMS 50L instrument (AMETEK, Cameca) detecting 7 secondary molecular ion species: $^{16}O^-$, $^{12}C_2^-$, $^{13}C^{12}C^-$, $^{12}C^{14}N^-$, $^{13}C^{14}N^-$, $^{32}S^-$ and $^{31}P^{16}O_2^-$. For the optimization, two main parameters were tuned: primary ion (PI) current and analyzed area size. The ability to focus the PI beam down to 30 nm resolution is usually achieved with the reduction of the beam current. In the present study, the nanoSIMS performance has been tested with 2 different values of PI current: 2 and 4 pA in order to clarify the possibility of speeding-up the measurement and enhancing counting statistics with higher secondary ion counts compromising the focus quality. Different sizes of analysis area were checked to reach an optimal pixel-count statistics for each single-cell-RoI with an acceptable number of single-cells resolved in the analyzed Field of View (FoV).
Prior to analysis, filter areas of 100×100 μm^2 were pre-implanted with primary ions beam Cesium (Cs^+) at 100 pA for 5 minutes. The number of pixels per single-cell can be tuned by changing the size of analyzed area keeping constant the raster size (512×512 pixels); therefore, 40×40, 30×30 and 20×20 µm² area were analyzed for the optimization. The dwell time was fixed to 2 ms/pixel for the whole optimization procedure.
The settings with 2 pA PI current was tested (Fig. S1) first to verify the influence of pixel number per single cell on the precision of single-cell derived isotope fraction. The best precision of isotope fraction at single-cell level was achieved for 2 pA PI current with 20×20 µm² analysis area (0.44±0.06%, mean of single-cell σ over isotope-fraction for all single-cells measured) due to maximal number of pixels per single-cell obtained with the smallest pixel size (39 nm versus 58 nm and 78 nm with 30 µm and 40 µm raster, respectively). With 2 pA PI current and 40×40 µm² analysis area, the relative error of single-cell isotope fraction reached 1.39±0.24%; therefore, this size of analysis area was not considered for further optimization. For the 30×30 µm² analysis area, the relative error was 0.79±0.16% with 2 pA PI current.
PI beam current of 4 pA (Fig. S2) with 30×30 µm² analysis area resulted in 0.88±0.14% precision of isotope fraction at single-cell level. The analysis settings with 4 pA PI current and 20×20 µm² analysis area were selected providing a considerable analysis speed and 0.37±0.05% relative error of isotopic content at single-cell level. In this condition, the highest pixel density with sufficient secondary ion counts per pixel (consequently better ion-count statistics for single-cells) and a considerable quality of PI beam focusing were achieved.
NanoSIMS data acquired for the optimization were processed with LANS (Polerecky et al., 2012); the single-cell RoI were drawn manually with the $^{12}C^{14}N^-$ map representing the biomass distribution.

The ^{13}C fraction ($D_{^{13}C}\times 100$ [at%]) was evaluated from the ratio of C_2^- molecular ion counts ($R = N_{^{13}C\,^{12}C}/N_{^{12}C^{12}C}$) using the following expression:

$$D_{^{13}C} = \frac{N_{^{13}C^{12}C}/N_{^{12}C^{12}C}}{\{2\times(N_{^{13}C^{12}C}/N_{^{12}C^{12}C})+1\}} = \frac{R_{C_2}}{\{2\times(R_{C_2}+1)\}}.$$

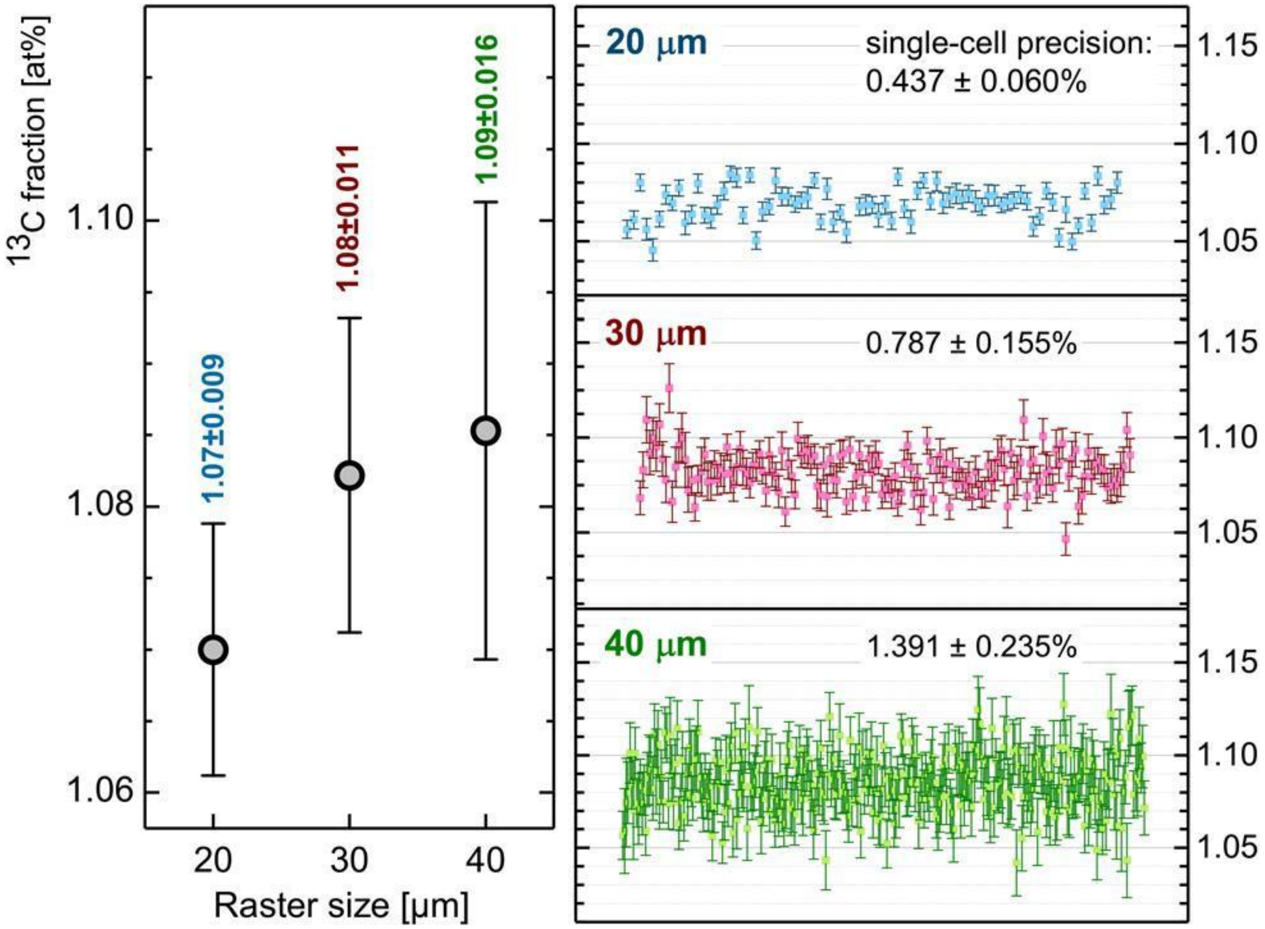

Supplementary Figure S1. Single-cell analysis of *P. stutzeri* cells. On the left panel mean value ± SD achieved with three different sizes of sample areas analyzed with 2 pA primary ion current are shown. On the right panel, single-cell values of ^{13}C content are shown; symbols represent the ^{13}C fraction [at%] and its error (±σ, error bar) for each single-cell.

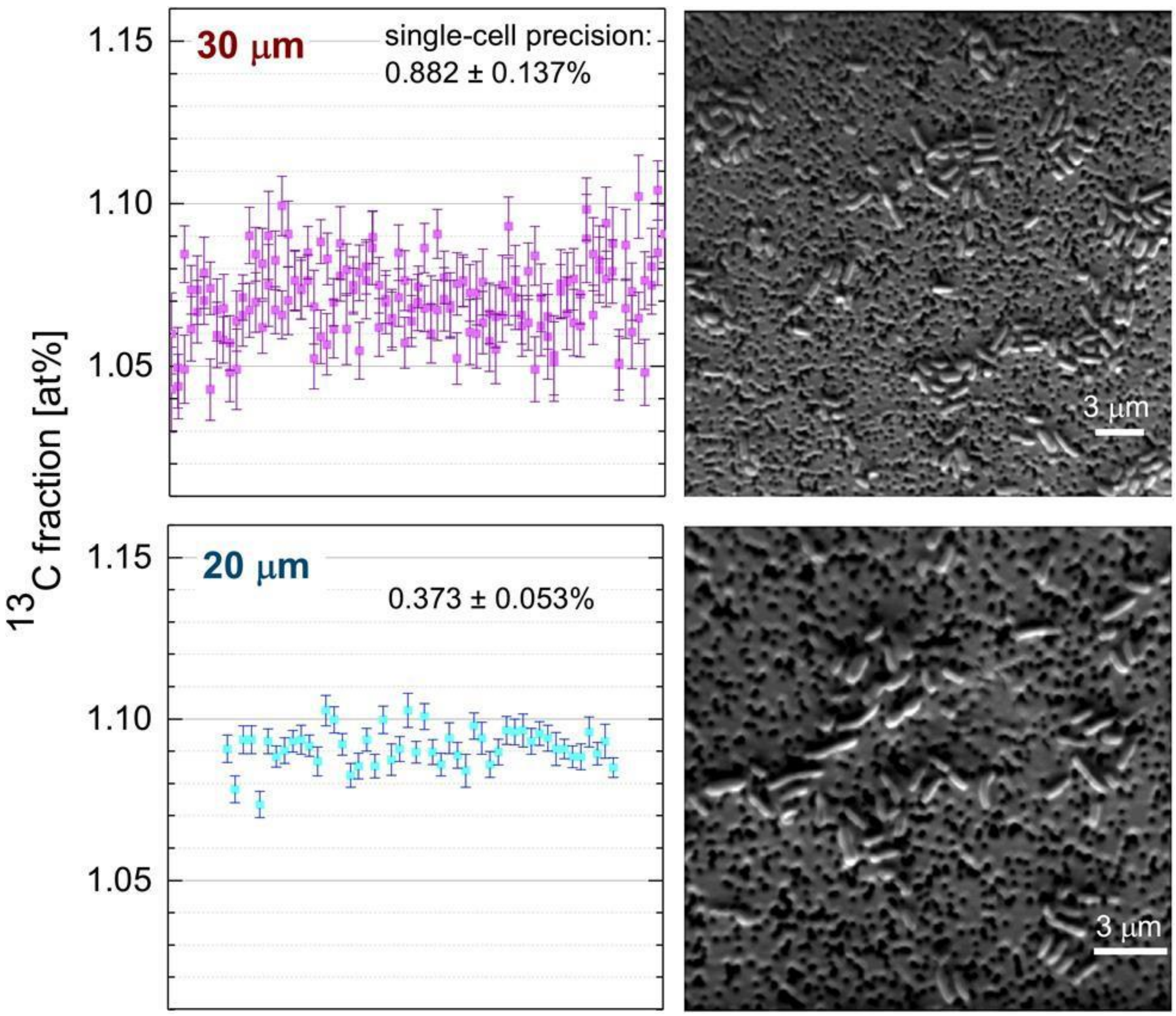

Supplementary Figure S2. Comparison of single-cell values of ^{13}C content and focus quality for 30x30 µm² and 20x20 µm² sample area (FoV) analyzed with 4 pA current of the primary Cs^{+} ion beam.

2. Zipfian fit of normally-distributed data

The approximation of an experimental rank-activity distribution with $K_A(r;\ q,\ s,\ d,\ n)$ (Eq. 25 in the main text) provides a slope value s (Differentiation Tendency Index, DTI) characterizing the tendency of single-cell to differentiate in their anabolic activity. It has been assumed that when the rank-activity distribution of all cells inside a monoclonal population fit a single slope, then the cells follow unimodal activity. To prove the feasibility to measure heterogeneity in cellular activity with the slope of rank-activity distribution, simulated normal distributions (Figs. S3-A, S4) were approximated with the Zipfian function (Eq. 25).

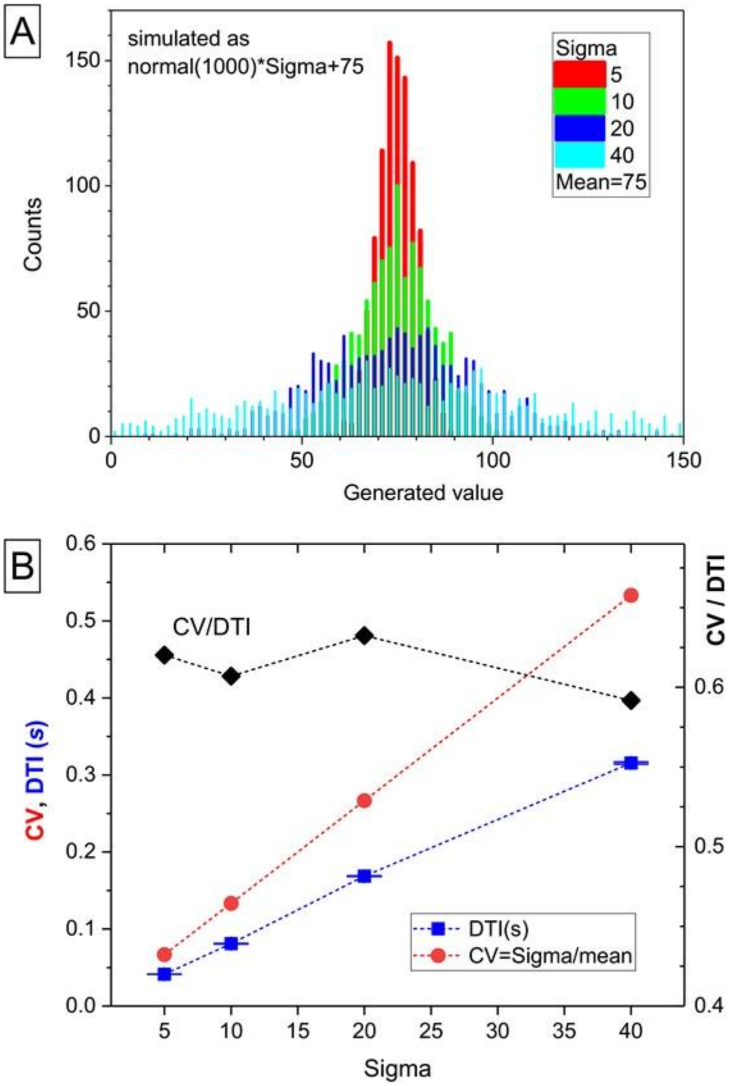

Supplementary Figure S3. Frame A: normal distribution simulated with fixed mean and different Sigma values. Frame B shows the DTI values derived as rank distribution slope with Zipfian fit ($s\ \pm\ \Delta s$, blue rectangles), corresponding CV values (red circles) and CV/DTI ratio calculated for different σ values with fixed the centroid (mean value) fixed at 75.

2.1. Correlation between Zipfian slope (DTI, *s*) and Coefficient of Variation (CV)

The DTI values, derived for the simulated normal distribution with fixed mean and different σ (Sigma; Standard Deviation, SD), were shown to reproduce the trend of corresponding CV values (Fig. S3-B) providing a proof for DTI as heterogeneity index.

2.2. Invariance of Zipfian slope to population size

Normal cellular activity distributions were simulated with different population size (1000, 200 and 50 cells; Fig. S4) and the rank-activity distribution of simulated data were approximated with Zipfian function (Fig. S5). OriginPro 2017 software was used for data simulation and approximation of rank-activity distributions.

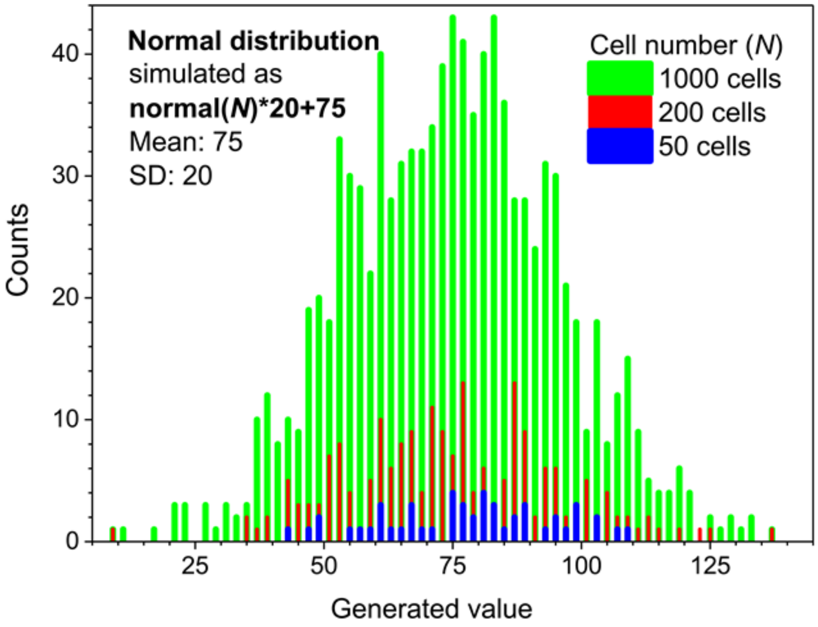

Supplementary Figure S4. Histograms plot of simulated normally distributed populations in single cell anabolic activity. The mean value and the SD (σ) were kept the same for different population size.

The approximation delivers the slope values (tables in Fig. S5) *s*=0.1624±0.0006 for 1000 cells, *s*=0.1631±0.0011 for 200 cells and *s*=0.1619±0.0082 for 50 cells proving the invariance of *s* to the population size.

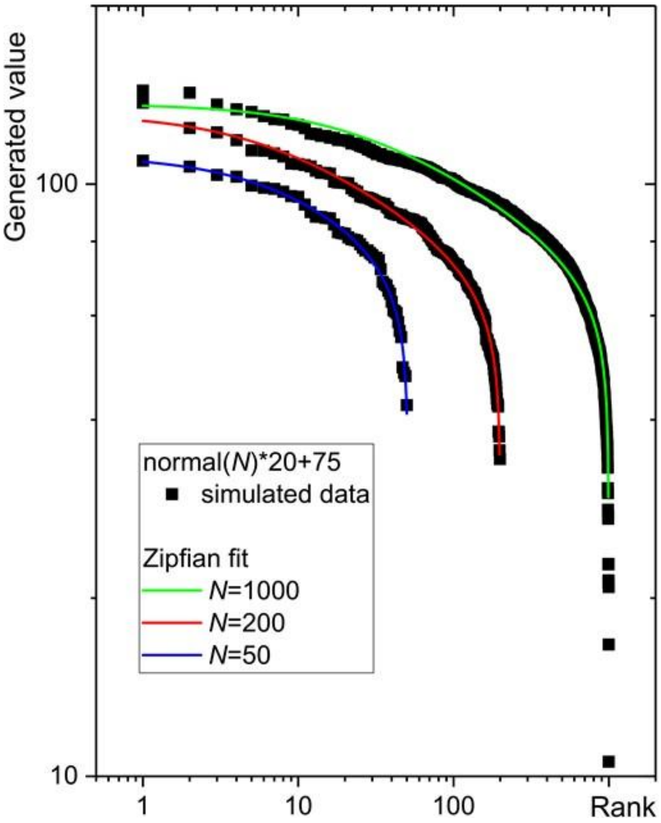

Model	Zipf (User)
Equation	C*exp(-s*Ln(n*(x+q)/(n-(x+q)+1)))
C	227.26313 ± 0.88611
q	22.57743 ± 0.32172
s	0.16236 ± 6.00718E-4
n	1018.35137 ± 0.19316
Reduced Chi-Sqr	4.87759
R-Square(COD)	0.98851
Adj. R-Square	0.98848

Model	Zipf (User)
Equation	C*exp(-s*Ln(n*(x+q)/(n-(x+q)+1)))
C	175.31763 ± 0.93637
q	5.7219 ± 0.0264
s	0.16306 ± 0.00115
n	204.72228 ± 0.35636
Reduced Chi-Sqr	2.26408
R-Square(COD)	0.9943
Adj. R-Square	0.99421

Model	Zipf (User)
Equation	C*exp(-s*Ln(n*(x+q)/(n-(x+q)+1)))
C	156.46815 ± 7.16729
q	7.12479 ± 1.5033
s	0.16189 ± 0.00824
n	56.95222 ± 1.94179
Reduced Chi-Sqr	1.82409
R-Square(COD)	0.99371
Adj. R-Square	0.9933

Supplementary Figure S5. Zipfian approximation of rank-activity distributions of cellular activity simulate for different population size (1000, 200 and 50 cells).

3. Zipfian approximation of rank-activity distribution

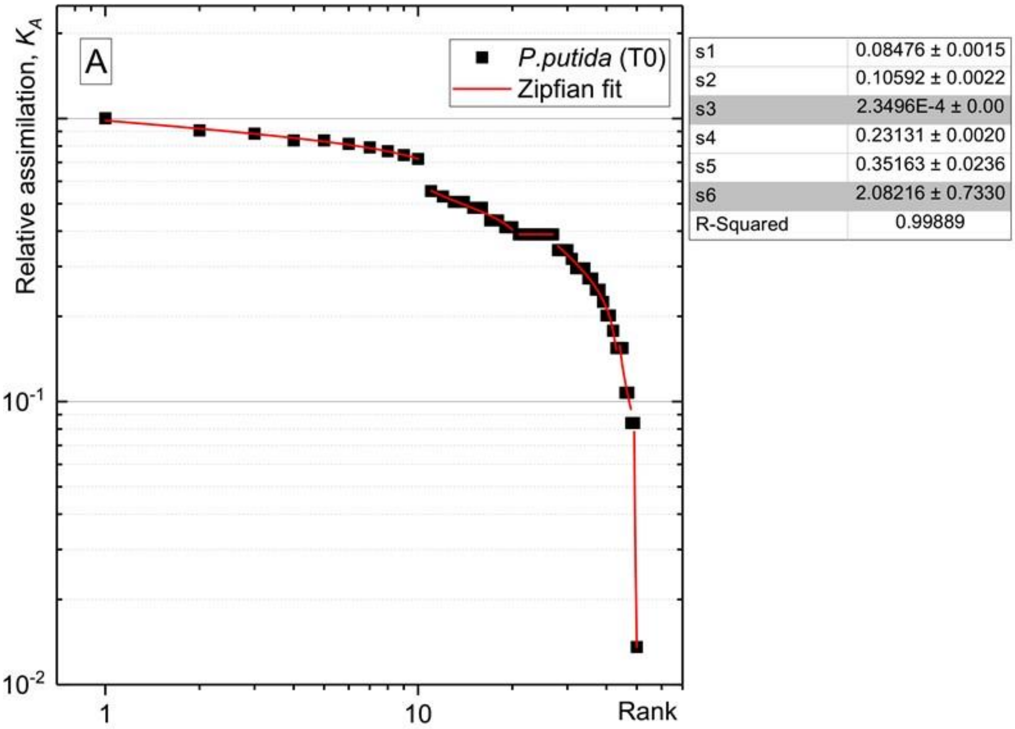

s1	0.08476 ± 0.0015
s2	0.10592 ± 0.0022
s3	2.3496E-4 ± 0.00
s4	0.23131 ± 0.0020
s5	0.35163 ± 0.0236
s6	2.08216 ± 0.7330
R-Squared	0.99889

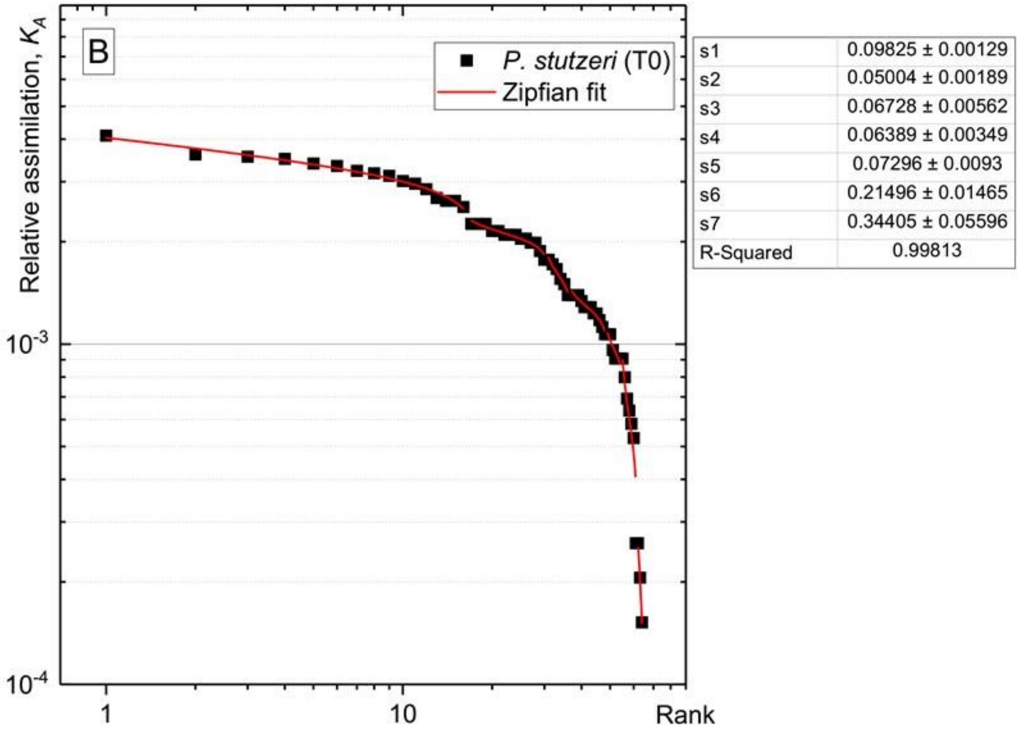

s1	0.09825 ± 0.00129
s2	0.05004 ± 0.00189
s3	0.06728 ± 0.00562
s4	0.06389 ± 0.00349
s5	0.07296 ± 0.0093
s6	0.21496 ± 0.01465
s7	0.34405 ± 0.05596
R-Squared	0.99813

Supplementary Figure S6. Rank-activity distributions and multi-component Zipfian approximation of experimental data. Rank-activity distributions of *P. putida* (frame A) and *P. stutzeri* (frame B) at Time 0 approximated with multi-component Zipfian function (Eq. 26 in the main text). The tables on the right side show the fitted slope values ($s \pm \Delta s$) and R-squared representing fit accuracy.

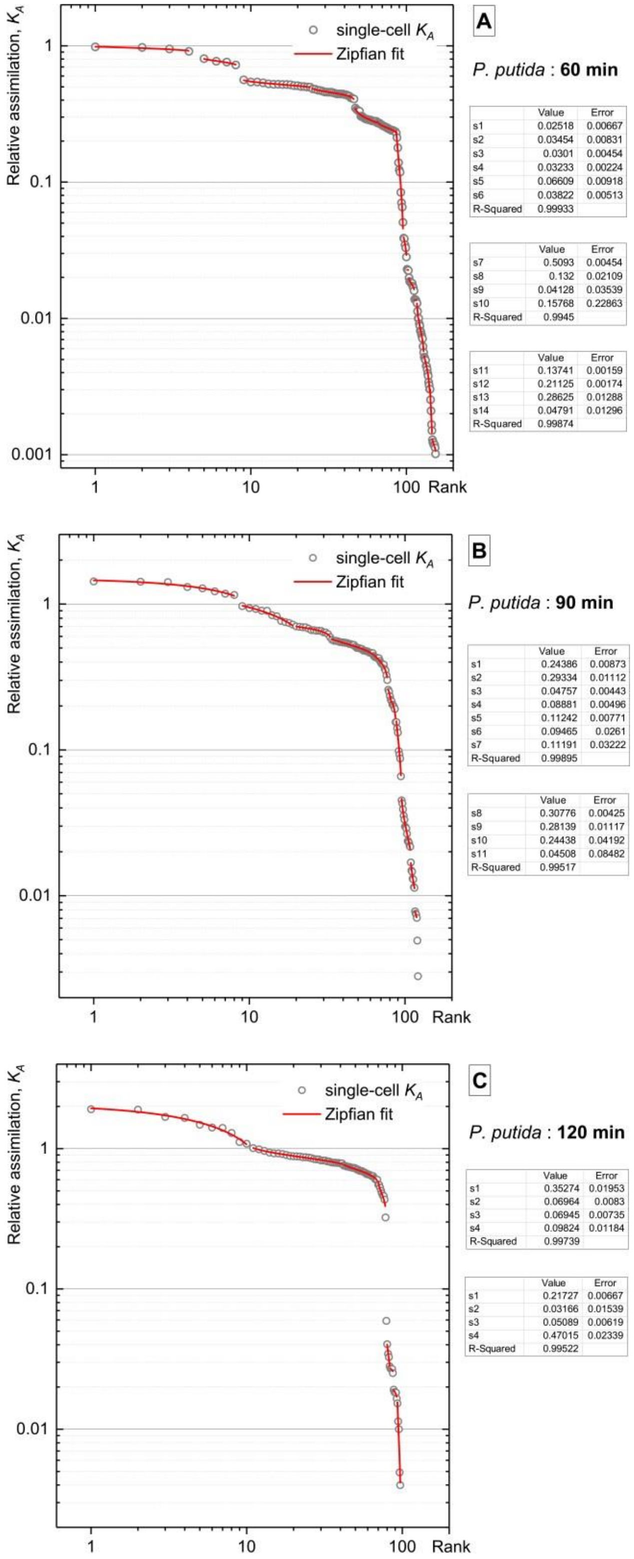

P. putida : **60 min**

	Value	Error
s1	0.02518	0.00667
s2	0.03454	0.00831
s3	0.0301	0.00454
s4	0.03233	0.00224
s5	0.06609	0.00918
s6	0.03822	0.00513
R-Squared	0.99933	

	Value	Error
s7	0.5093	0.00454
s8	0.132	0.02109
s9	0.04128	0.03539
s10	0.15768	0.22863
R-Squared	0.9945	

	Value	Error
s11	0.13741	0.00159
s12	0.21125	0.00174
s13	0.28625	0.01288
s14	0.04791	0.01296
R-Squared	0.99874	

P. putida : **90 min**

	Value	Error
s1	0.24386	0.00873
s2	0.29334	0.01112
s3	0.04757	0.00443
s4	0.08881	0.00496
s5	0.11242	0.00771
s6	0.09465	0.0261
s7	0.11191	0.03222
R-Squared	0.99895	

	Value	Error
s8	0.30776	0.00425
s9	0.28139	0.01117
s10	0.24438	0.04192
s11	0.04508	0.08482
R-Squared	0.99517	

P. putida : **120 min**

	Value	Error
s1	0.35274	0.01953
s2	0.06964	0.0083
s3	0.06945	0.00735
s4	0.09824	0.01184
R-Squared	0.99739	

	Value	Error
s1	0.21727	0.00667
s2	0.03166	0.01539
s3	0.05089	0.00619
s4	0.47015	0.02339
R-Squared	0.99522	

Supplementary Figure S7. Rank-activity distributions and multi-component Zipfian approximation for *P. putida* at different time points.

When cells reveal multimodal activity, their rank-activity distribution reveals steps assigned to subpopulations belonging to the same monoclonal population. Fit of a multimodal rank activity distribution with single-component Zipfian function (Eq. 25 in the main text) results in large relative [%]) error in slope value ($\Delta s/s \times 100$ [%]) 1.9%, 17.2%, 3.7% for the 15, 30 and 60 minutes incubation time, respectively; Fig. S8). In such a case the multi-component Zipfian function has to be applied for data fit and CDTI ($S \pm \Delta S$, Eqs. 27, 28 in the main text) has to be derived to quantify the heterogeneity of entire population.

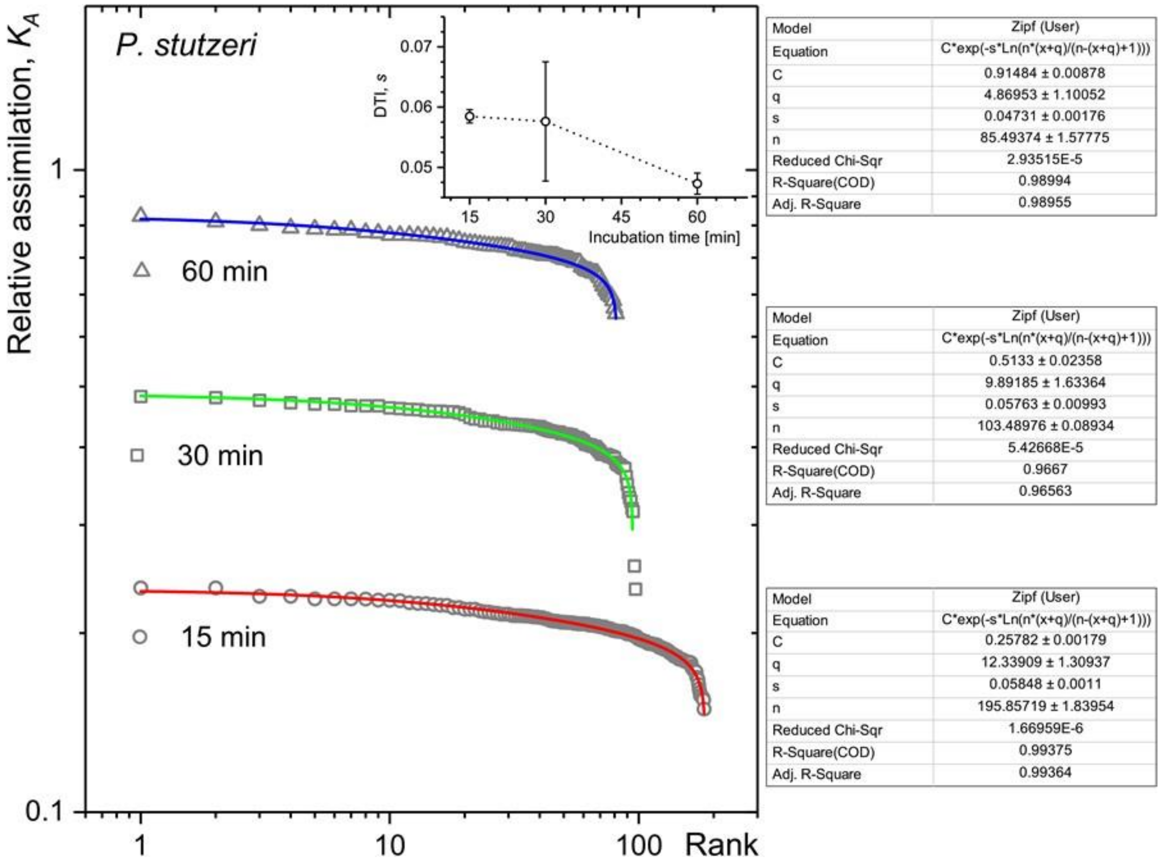

Model	Zipf (User)
Equation	C*exp(-s*Ln(n*(x+q)/(n-(x+q)+1)))
C	0.91484 ± 0.00878
q	4.86953 ± 1.10052
s	0.04731 ± 0.00176
n	85.49374 ± 1.57775
Reduced Chi-Sqr	2.93515E-5
R-Square(COD)	0.98994
Adj. R-Square	0.98955

Model	Zipf (User)
Equation	C*exp(-s*Ln(n*(x+q)/(n-(x+q)+1)))
C	0.5133 ± 0.02358
q	9.89185 ± 1.63364
s	0.05763 ± 0.00993
n	103.48976 ± 0.08934
Reduced Chi-Sqr	5.42668E-5
R-Square(COD)	0.9667
Adj. R-Square	0.96563

Model	Zipf (User)
Equation	C*exp(-s*Ln(n*(x+q)/(n-(x+q)+1)))
C	0.25782 ± 0.00179
q	12.33909 ± 1.30937
s	0.05848 ± 0.0011
n	195.85719 ± 1.83954
Reduced Chi-Sqr	1.66959E-6
R-Square(COD)	0.99375
Adj. R-Square	0.99364

Supplementary Figure S8. Rank-activity distributions and single-component Zipfian approximation for *P. stutzeri* at different time points. The tables show the results of single-component Zipfian fit. The inset shows the $s \pm \Delta s$ obtained with the fit at different time points.

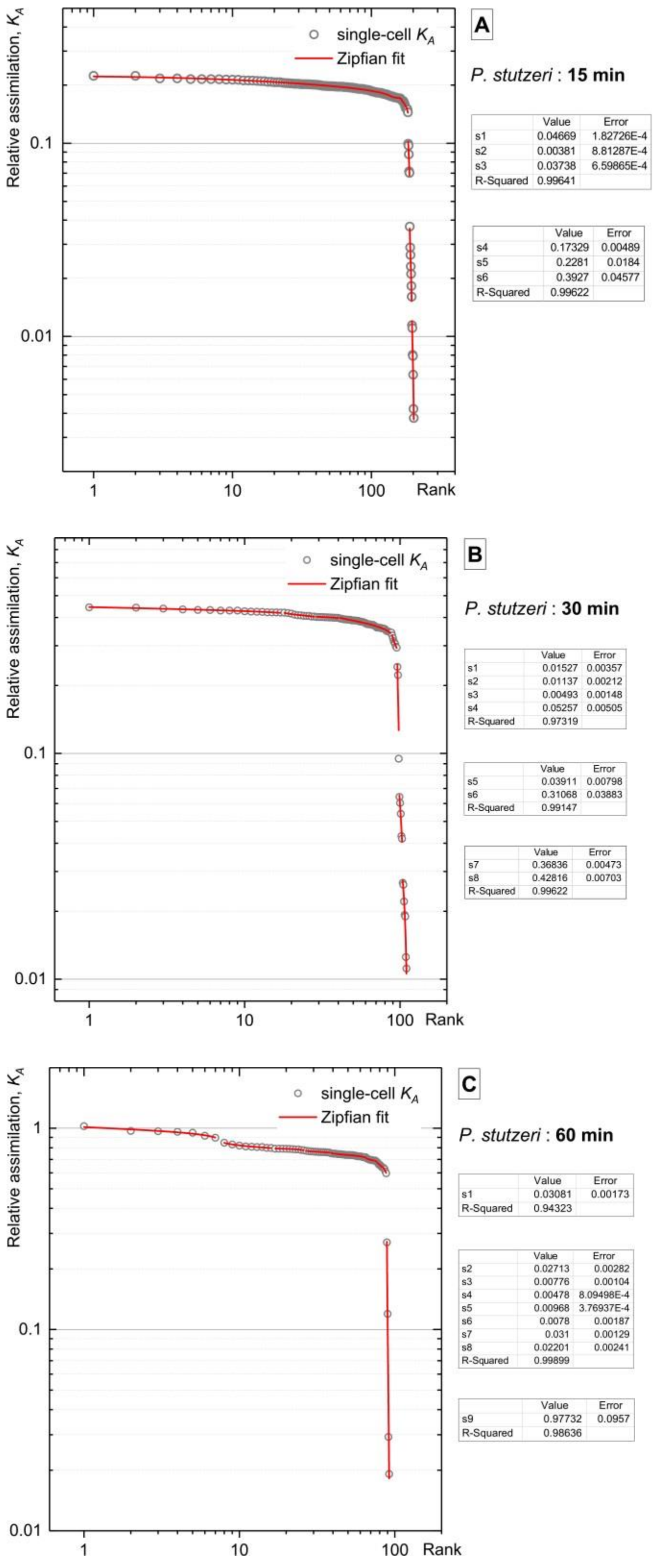

Supplementary Figure S9. Rank-activity distributions and multi-component Zipfian approximation for *P. stutzeri* at different time points. At time point 60 minutes (frame C), 4 cells were initially assigned to a low-SDT subpopulation. However, such cells, distributed over one order of magnitude of their K_A values, could not be further considered as a single subpopulation and were therefore

excluded from the calculation of the corresponding CDTI value.

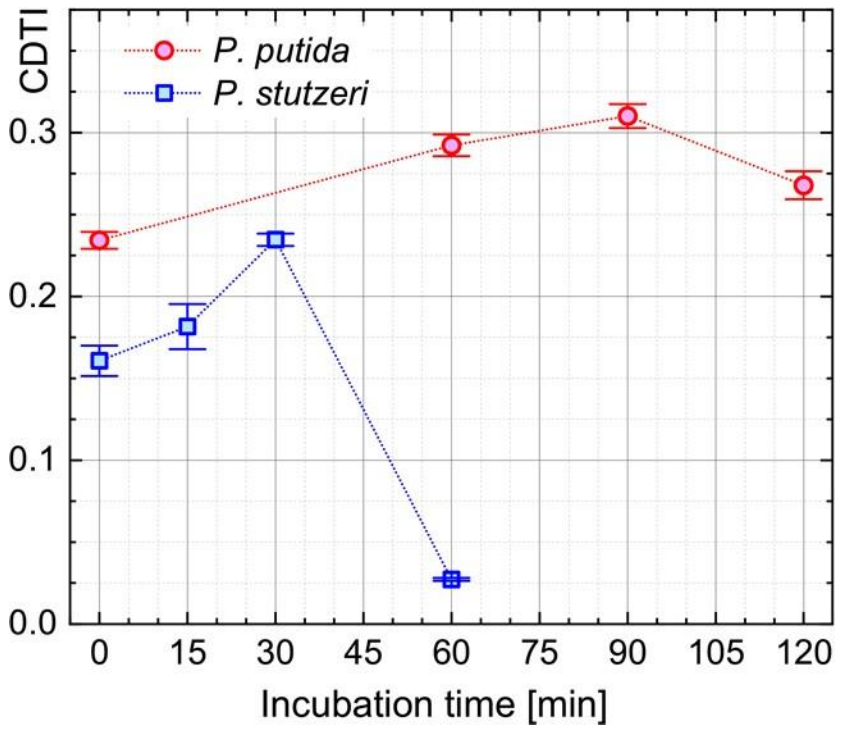

Supplementary Figure S10. The comparison of heterogeneity in anabolic activity of *P. putida* and *P. stutzeri* strains represented as CDTI trends over time.

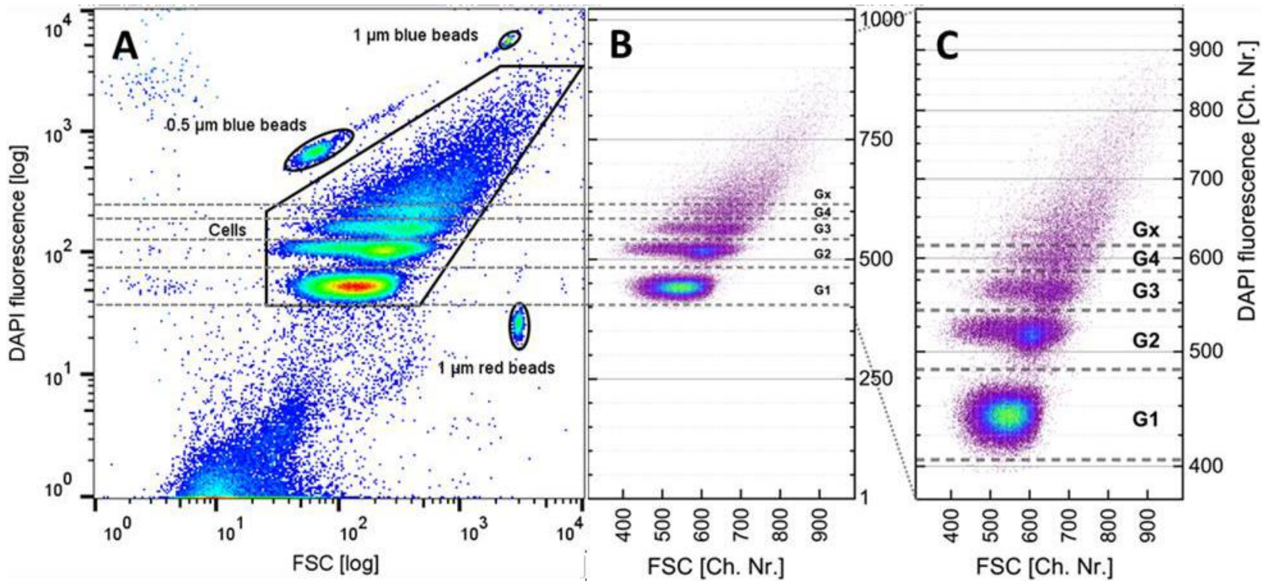

Supplementary Figure S11. Transformation of flow cytometric data prior to Zipfian approximation. The procedure was exemplarily visualized on the data acquired on *P. putida* cells at 0 h time point. 10^5 DAPI-stained cells were measured in logarithmic amplification mode and visualized in a forward scatter intensity (FSC, related to cell size) vs. DAPI fluorescence intensity (related to DNA content) dot plot (Frame A). Standard particle beads were measured with every sample to secure instrument stability. The events in the cell-gate were exported from the original .fsc files with the logarithmic scales of both axis evenly distributed into 1024 channels ranging from 0 to 1023 (Frame B). The boundaries between the five gates G1 to Gx were defined according to the local minima in the DAPI- fluorescence intensity distribution. In the last step, the range of DAPI-fluorescence intensity scale was confined within the cell-containing channels (channels: 375 to 975). This range was then represented on a warped scale to enhance resolution of the lower channel numbers where the majority of cells where measured (Frame C).

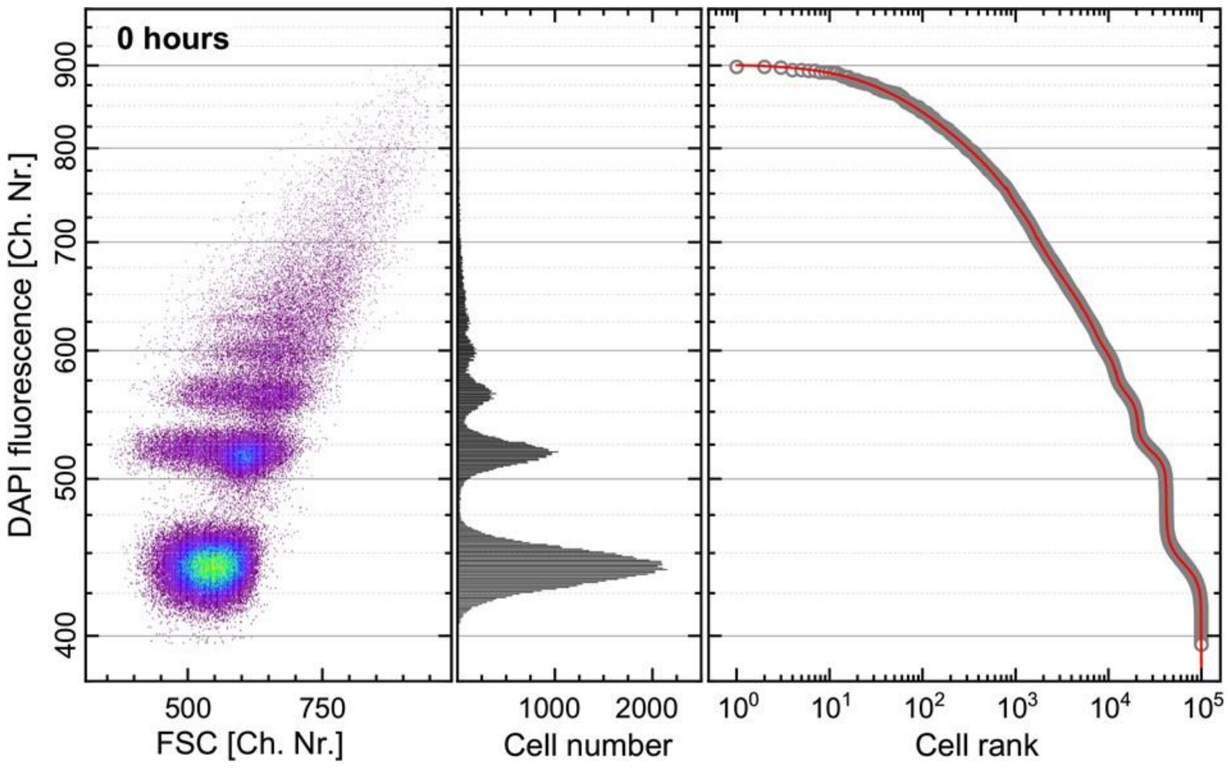
0 hours
DAPI fluorescence [Ch. Nr.]
900
800
700
600
500
400
500
750
FSC [Ch. Nr.]
1000
2000
Cell number
10^0
10^1
10^2
10^3
10^4
10^5
Cell rank

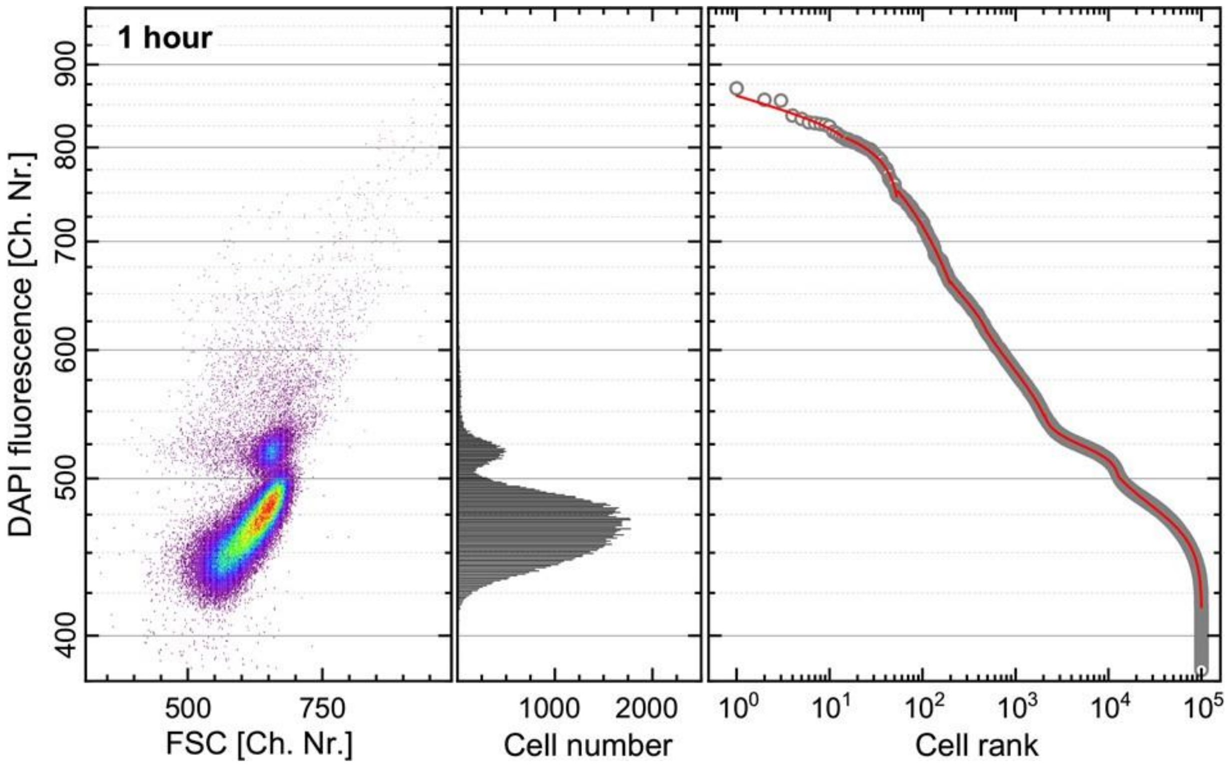
1 hour
DAPI fluorescence [Ch. Nr.]
900
800
700
600
500
400
500
750
FSC [Ch. Nr.]
1000
2000
Cell number
10^0
10^1
10^2
10^3
10^4
10^5
Cell rank

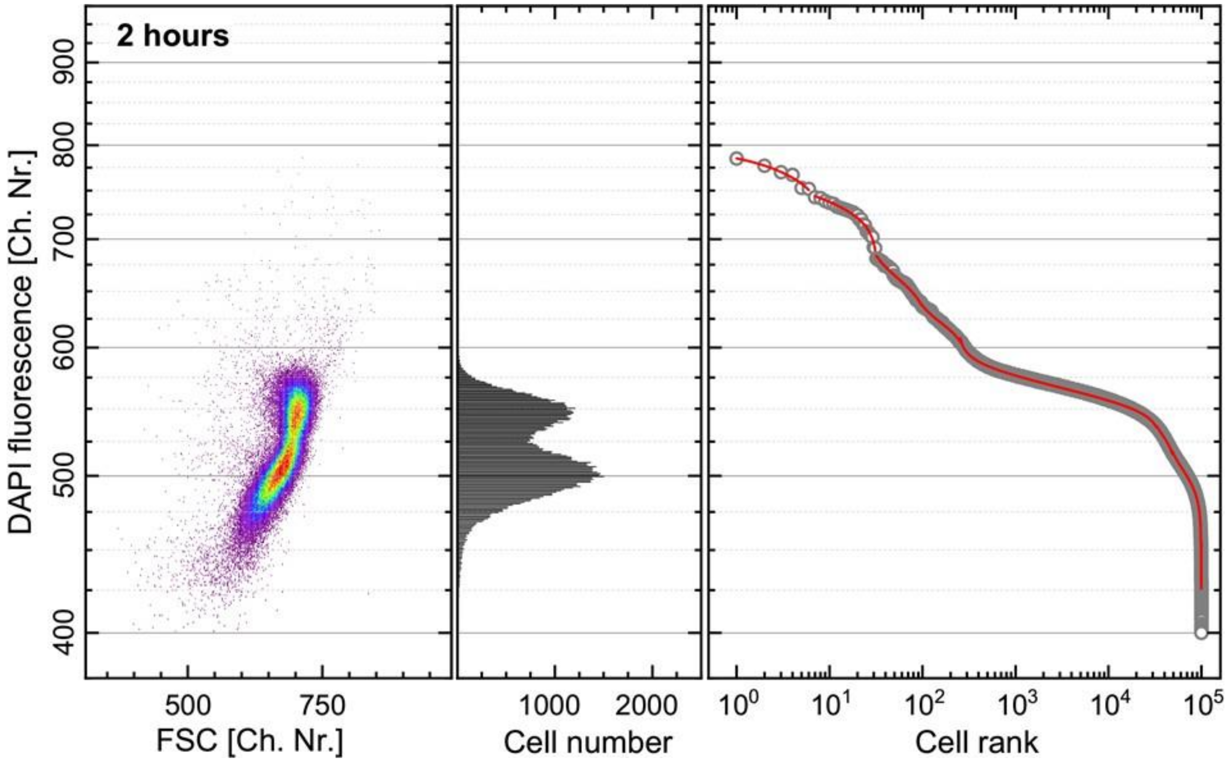
2 hours
DAPI fluorescence [Ch. Nr.]
400
500
600
700
800
900
500
750
FSC [Ch. Nr.]
1000
2000
Cell number
10^0
10^1
10^2
10^3
10^4
10^5
Cell rank

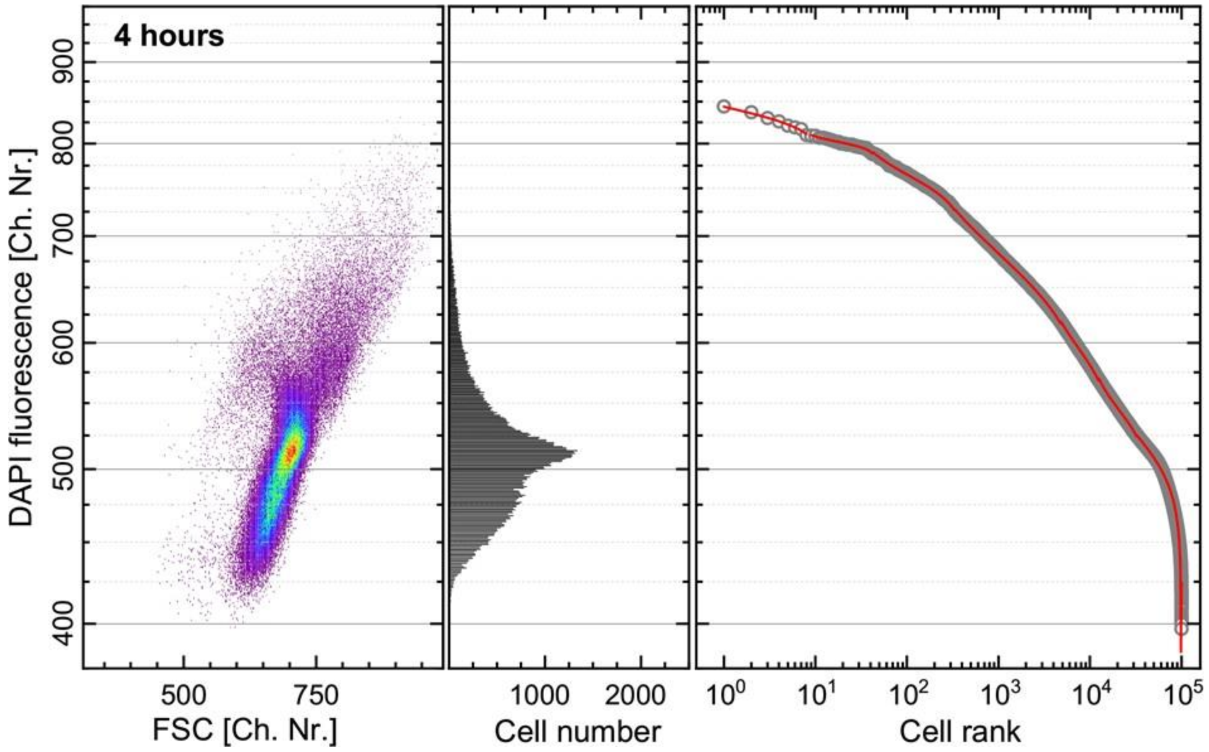
4 hours
DAPI fluorescence [Ch. Nr.]
400
500
600
700
800
900
500
750
FSC [Ch. Nr.]
1000
2000
Cell number
10^0
10^1
10^2
10^3
10^4
10^5
Cell rank

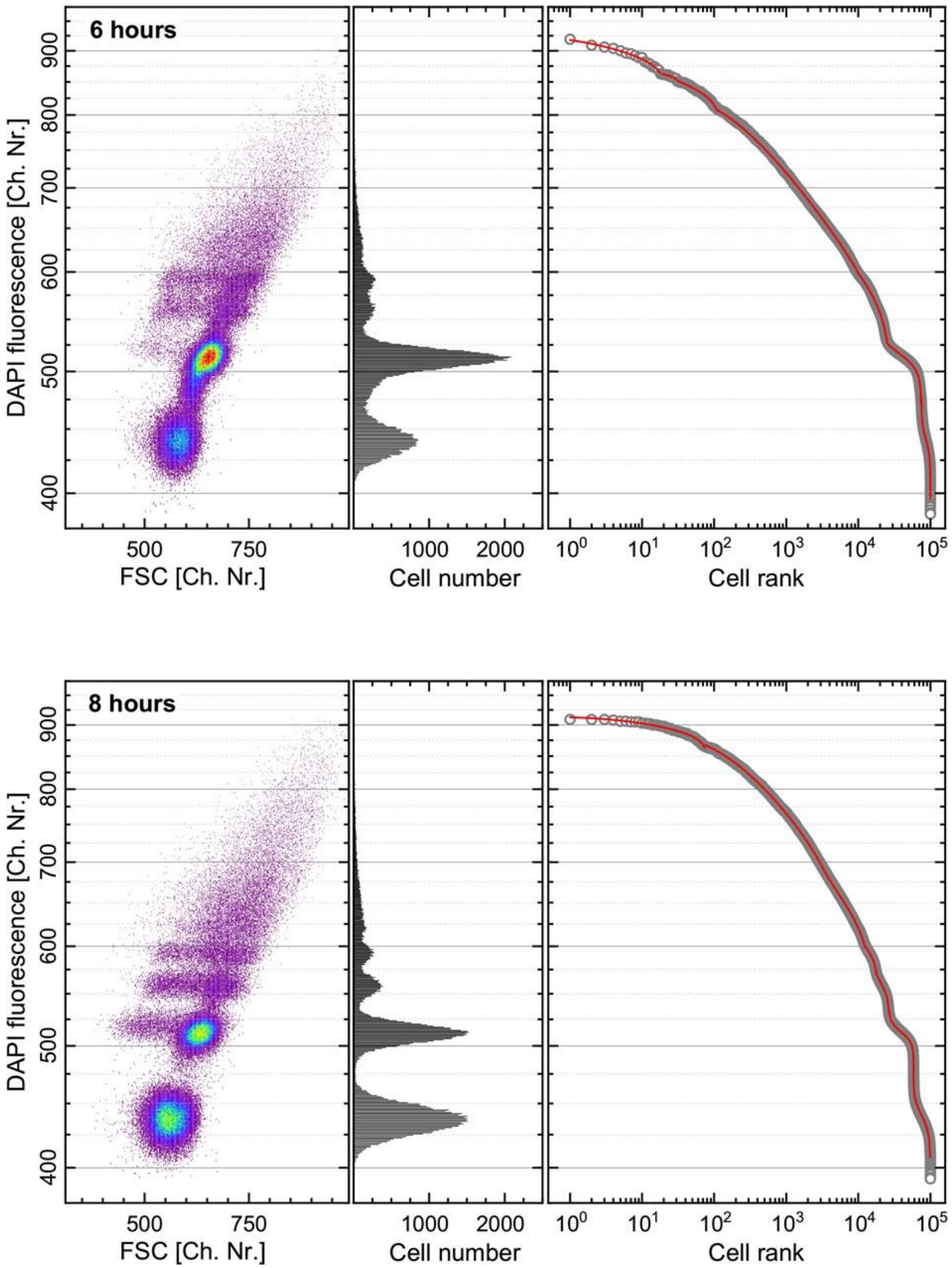

6 hours
DAPI fluorescence [Ch. Nr.]
400
500
600
700
800
900
500
750
FSC [Ch. Nr.]
1000
2000
Cell number
10^0
10^1
10^2
10^3
10^4
10^5
Cell rank
8 hours
DAPI fluorescence [Ch. Nr.]
400
500
600
700
800
900
500
750
FSC [Ch. Nr.]
1000
2000
Cell number
10^0
10^1
10^2
10^3
10^4
10^5
Cell rank

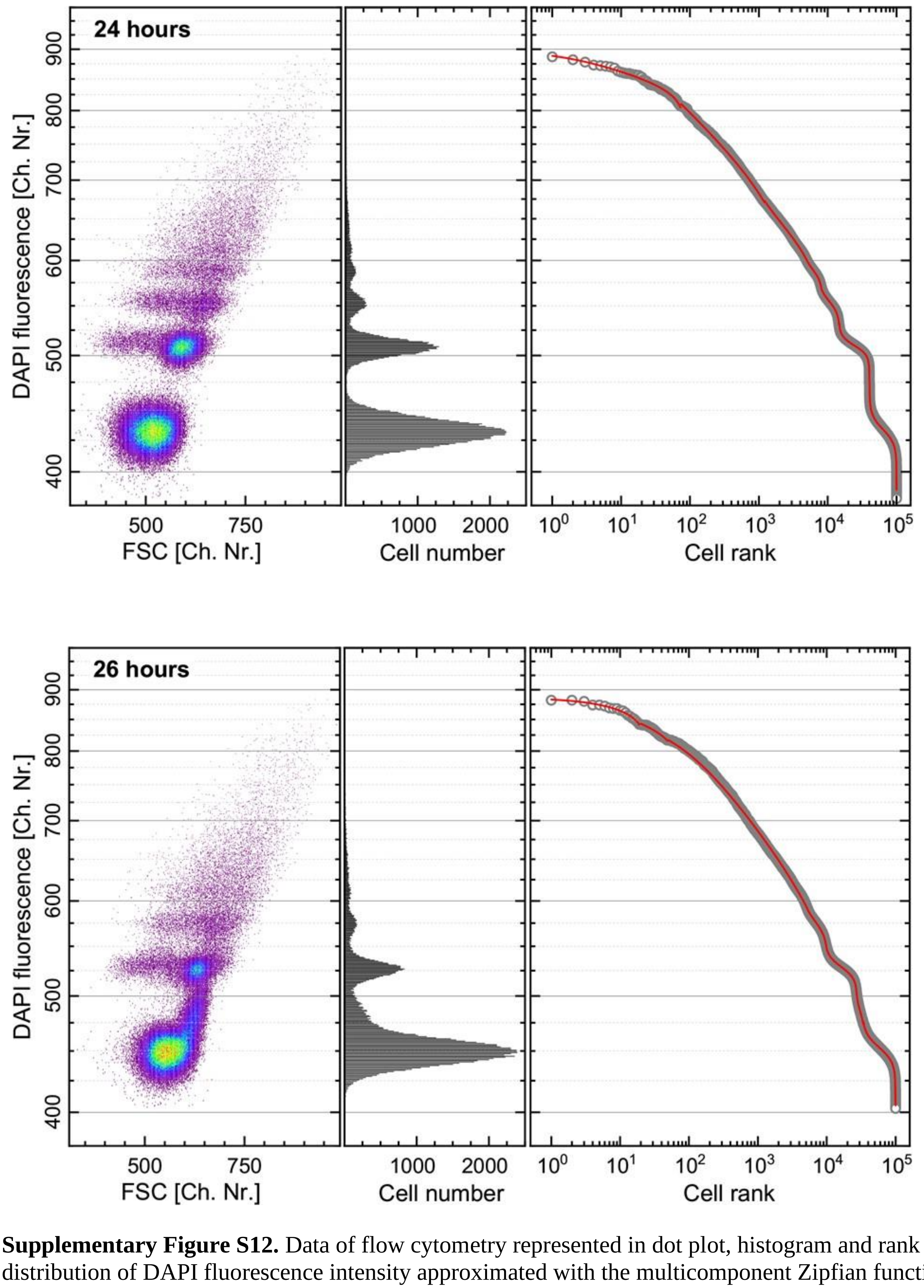

Supplementary Figure S12. Data of flow cytometry represented in dot plot, histogram and rank distribution of DAPI fluorescence intensity approximated with the multicomponent Zipfian function.
